# Supplementary material for: Clinical efficacy and safety of automatic remifentanil administration based on Analgesia Nociception Index monitoring during burn surgery under propofol anesthesia: A randomized controlled clinical trial
Source: PLoS One. 2025 May 5;20(5):e0322384. doi: 10.1371/journal.pone.0322384 (PMC12052174; doi:10.1371/journal.pone.0322384)
Supplement: S1 File — (DOCX) [file pone.0322384.s001.docx]

**Table 5.** Secondary outcomes during anesthesia (per protocol analysis)

|  | **Standard Practice Group**  ***(n=24)*** | **Automatic Group (*n* = 26)** | **Effect size (95% CI)** | ***P* value** |
| --- | --- | --- | --- | --- |
| Administration of Ephedrine | 6 (25.0) | 6 (23.1) | 0.92 (0.34–2.48)^7^ | 0.87 |
| Total dose of Ephedrine administered (mg) | 9 (6–12)^1^ | 9 (6–12)^1^ | - | - |
| Proportion of time spent in ANIi intervals (%) : |  |  |  |  |
| <50 | 17 (12–28)^3^ | 19 (11–36)^4^ | 0.21 (-0.36–0.78) | 0.48 |
| [50 ;70] | 41 (28–47)^3^ | 39 (29–43)^4^ | -0.22 (-0.79–0.35) | 0.45 |
| >70 | 40 (24–57)^3^ | 39 (21–54)^4^ | -0.07 (-0.64–0.50) | 0.81 |
| Proportion of time spent in ANIa intervals (%) : |  |  |  |  |
| <50 | 15 (7–22)^3^ | 15 (8–27)^4^ | 0.09 (-0.48–0.66) | 0.76 |
| [50 ; 70] | 48 (27–59)^3^ | 46 (32–54)^4^ | -0.04 (-0.61–0.53) | 0.90 |
| >70 | 34 (18–60) | 36 (14–58)^4^ | -0.05 (-0.62–0.52) | 0.87 |
| Proportion of time spent in BIS intervals (%) : |  |  |  |  |
| <40 | 51 (15–69) | 51 (25–69)^4^ | 0.11 (-0.46–0.67) | 0.71 |
| [40 ; 60] | 49 (29–82) | 37 (24–71)^4^ | -0.24 (-0.81–0.33) | 0.41 |
| >60 | 2 (0–4) | 2 (0–7)^4^ | 0.24 (-0.33–0.80) | 0.41 |
| Cumulative propofol dose during anesthesia (mg.kg^-1^.min^-1^) | 0.113 (0.094–0.129) | 0.115 (0.098–0.162) | 0.47 (-0.09–1.04) | 0.11 |
| Number of propofol target changes | 4 (3–10) | 4 (2–8) | 1.17 (0.71–1.94)^9^ | 0.53 |

Values are number (%), median (25th to 75th percentile) or mean ± standard deviation. Effect sizes are standardized differences except for ^1^ relative risk and ^2^ risk ratio. CI = confidence interval;
