# Supplementary material for: Clinical efficacy and safety of automatic remifentanil administration based on Analgesia Nociception Index monitoring during burn surgery under propofol anesthesia: A randomized controlled clinical trial
Source: PLoS One. 2025 May 5;20(5):e0322384. doi: 10.1371/journal.pone.0322384 (PMC12052174; doi:10.1371/journal.pone.0322384)
Supplement: S2 File — (DOCX) [file pone.0322384.s002.docx]

**Table 6.** Secondary outcomes two hours after anesthesia (per protocol analysis)

|  | **Standard Practice Group**  ***(n=24)*** | **Automatic Group (*n* = 26)** | **Effect size (95% CI)** | ***P* value** |
| --- | --- | --- | --- | --- |
| VAS, H0+15min | 60 (40–70)^3^ | 60 (50–70) | 0.11 (-0.46–0.68) | 0.71 |
| VAS, H0+30min | 50 (40–70)^3^ | 57 (33–70) | -0.05 (-0.62–0.51) | 0.86 |
| VAS, H0+45min | 40 (30–60)^3^ | 40 (20–50)^4^ | -0.37 (-0.95–0.20) | 0.21 |
| VAS, H0+1h | 38 (30–40) | 30 (10–43)^4^ | -0.46 (-1.03–0.11) | 0.12 |
| VAS, H0+1h15min | 30 (20–40)^3^ | 20 (11–40)^4^ | -0.21 (-0.79–0.36) | 0.47 |
| VAS, H0+1h30min | 20 (13–30) | 16 (10–30)^4^ | -0.33 (-0.89–0.24) | 0.26 |
| VAS, H0+1h45min | 19 (10–24) | 10 (10–20)^4^ | -0.31 (-0.87–0.26) | 0.29 |
| VAS, H0+2h | 15 (10–20) | 10 (0–20)^4^ | -0.12 (-0.69–0.44) | 0.67 |
| Cumulative morphine dose administered (mg) | 9 (4–12) | 10 (5–12) | 0.15 (-0.41–0.71) | 0.60 |
| Administration of ketamine | 7 (29.2) | 10 (38.5) | 1.32 (0.59–2.91)^7^ | 0.49 |
| Total ketamine dose administered (mg) | 20 (20–20)^5^ | 20 (20–20)^6^ | - | - |
| Nausea | 0 | 1 (3.9) | NA | NA |
| Vomiting | 0 | 0 | NA | NA |

Values are number (%), median (25th to 75th percentile) or mean ± standard deviation. Effect sizes are standardized differences except for ^1^ relative risk and ^2^ risk ratio. VAS = Visual Analog Scale for pain ; CI = confidence interval ; NA = not applicable
