# Supplementary material for: Clinical efficacy and safety of automatic remifentanil administration based on Analgesia Nociception Index monitoring during burn surgery under propofol anesthesia: A randomized controlled clinical trial
Source: PLoS One. 2025 May 5;20(5):e0322384. doi: 10.1371/journal.pone.0322384 (PMC12052174; doi:10.1371/journal.pone.0322384)
Supplement: S4 File — (DOCX) [file pone.0322384.s004.docx]

« **Pilotage automatique de l’administration de remifentanil guidé par l’ANI au cours de l’anesthésie générale au propofol »**

# ANI-REMI-loop

N° de code attribué par le promoteur : **2016_48**

N° IDRCB : **2017-A00858-45**

| Recherche Impliquant la Personne Humaine (RIPH) | Recherche de catégorie 1 : Recherche Interventionnelle qui comporte une intervention sur la personne non justifiée par sa prise  en charge habituelle |
| --- | --- |

Investigateur Principal:

Dr JEANNE Mathieu

Clinique d’Anesthésie Réanimation Roger Salengro Boulevard Emile Laine

59037 LILLE cedex

Tél 03 20 44 40 74

Mail : [mathieu.jeanne@chru-lille.fr](mailto:mathieu.jeanne@chru-lille.fr)

Investigateurs :

Dr LAFANECHERE Aurélie

Clinique d’Anesthésie Réanimation Roger Salengro Boulevard Emile Laine

59037 LILLE cedex

tél 03 20 44 40 74

mail : [aurelie.lafanechere@chru-lille.fr](mailto:aurelie.lafanechere@chru-lille.fr)

Dr DEVAUCHELLE Pauline

Clinique d’Anesthésie Réanimation Roger Salengro Boulevard Emile Laine

59037 LILLE cedex

tél 03 20 44 40 74

mail : [Pauline.CLEMENTGRANDCOURT@CHRU-LILLE.FR](mailto:Pauline.CLEMENTGRANDCOURT@CHRU-LILLE.FR)

Collaborateur scientifique : DE JONCKHEERE Julien, Chargé de mission de recherche

INSERM CIC-IT 1403, MRRC

6 rue du professeur Laguesse 59037 LILLE Cedex

Tél 03 20 44 67 54

Mail : [julien.dejonckheere@chru-lille.fr](mailto:julien.dejonckheere@chru-lille.fr)

FLOCTEIL Mathilde, Technicienne d’Etude Clinique INSERM CIC-IT 1403, MRRC

6 rue du professeur Laguesse 59037 LILLE Cedex

Tél 0320446754

Mail : [mathilde.flocteil@chru-lille.fr](mailto:mathilde.flocteil@chru-lille.fr)

Promoteur de l’étude : **Centre Hospitalier Régional & Universitaire de Lille**

Département de la Recherche en Santé (DRS) 6 rue Paul Laguesse

59037 LILLE Cedex

tél : 03 20 44 59 69

N° de version en cours/Date : Version 2.0 du 19/01/2018

#### PAGE DE SIGNATURE DU PROTOCOLE

**Code promoteur :** 2016_48

**Titre long/court :** Pilotage automatique de l’administration de remifentanil guidé par l’ANI au cours de l’anesthésie générale au propofol / ANI-REMI-loop.

**N° de version et date :** n°2.0 du 19/01/2018

L’investigateur principal et le promoteur s’engagent à réaliser cette étude selon le protocole, les règles et les recommandations des bonnes pratiques cliniques internationales et selon les dispositions législatives et réglementaires applicables à la recherche.

| PROMOTEUR REPRESENTANT LEGAL | CHRU de Lille  Mr Frédéric BOIRON | DATE  _ _ / _ _ / _ _ _ _ | SIGNATURE |
| --- | --- | --- | --- |

#### SIGNATURE DE L’INVESTIGATEUR PRINCIPAL

J'ai lu l’ensemble des pages de ce protocole dont le CHRU de Lille est le promoteur, et je confirme qu'il contient toutes les informations nécessaires à la conduite de l’essai.

Je m'engage à réaliser l’essai en respectant le protocole et les termes et conditions qui y sont définis ainsi que ses éventuels amendements qui me seront transmis par le promoteur. Je m'engage à conduire ce protocole conformément aux Bonnes Pratiques Cliniques, à la Loi de Santé Publique du 9 août 2004 et du décret d’application du 16 novembre 2016 et notamment en délivrant l’information et en recueillant le consentement écrit des patients avant toute procédure de sélection du protocole.

Je m'engage à ce que les investigateurs et les autres membres qualifiés de mon équipe aient accès aux copies de ce protocole et des documents relatifs à la conduite de l’essai pour leur permettre de travailler dans le respect des dispositions figurant dans ces documents.

Je suis informé que mes données nominatives font l’objet d’un traitement automatisé ayant pour finalité la mise en place et le déroulement de la recherche. Ces informations peuvent éventuellement faire l’objet de transfert hors de l’Union Européenne. Conformément à la loi du 6 janvier 1978 modifiée, je bénéficie auprès du promoteur d’un droit d’accès et de rectification de mes informations personnelles

| INVESTIGATEUR PRINCIPAL ETABLISSEMENT | Dr Mathieu JEANNE  Clinique d’Anesthésie Réanimation Roger Salengro Boulevard Emile Laine 59037 LILLE cedex | DATE  _ _ / _ _ / _ _ _ _ | SIGNATURE |
| --- | --- | --- | --- |

# SOMMAIRE

[Liste des abréviations 5](#_bookmark0)

1. [SYNOPSIS 6](#_bookmark1)
2. [Rationnel : Justification scientifique et description générale de la recherche](#_bookmark2) [9](#_bookmark2)
3. [Objectif de la recherche 10](#_bookmark3)
   1. [Objectif principal 10](#_bookmark4)
   2. [Objectifs secondaires et les hypothèses générées 10](#_bookmark5)
4. [Conception de la recherche 10](#_bookmark6)
   1. [Plan expérimentale 10](#_bookmark7)
   2. [Définition de la population étudiée 11](#_bookmark8)
      1. [Critères d’inclusion 11](#_bookmark9)
      2. [Critères de non-inclusion 11](#_bookmark10)
      3. [Critère d’exclusion 12](#_bookmark11)
   3. [Critères d’évaluation 12](#_bookmark12)
      1. [Définitions 12](#_bookmark13)
      2. [critère principal, permettant de répondre à l’objectif principal 12](#_bookmark14)
      3. [critères secondaires permettant de répondre aux objectifs secondaires: 12](#_bookmark15)
   4. [Calcul du nombre de sujets 13](#_bookmark16)
   5. [Méthode et stratégie d’analyse 13](#_bookmark17)
5. [Logistique de l’étude 13](#_bookmark18)
   1. [Equipes participantes et expérience de l’équipe dans le domaine 13](#_bookmark19)
   2. [Déroulement pratique de la recherche 14](#_bookmark20)
      1. [Consentement éclairé 14](#_bookmark21)
      2. [Dispositifs utilisés pour l'anesthésie 14](#_bookmark22)
      3. [Déroulement de l'anesthésie 14](#_bookmark23)
   3. [Durée 16](#_bookmark24)
   4. [Critères d’arrêt de participation à l’étude 16](#_bookmark25)
   5. [Interdiction de participation simultanée – Période d’exclusion 16](#_bookmark26)
   6. [Bénéfices, risques et contraintes de l’étude 16](#_bookmark27)
      1. [BENEFICE 16](#_bookmark28)
      2. [BENEFICE COLLECTIF 16](#_bookmark29)
      3. [RISQUES 16](#_bookmark30)
      4. [Comité de Surveillance 16](#_bookmark31)
6. [Dispositif médical 17](#_bookmark32)
7. [Evaluation de la sécurité 17](#_bookmark33)
   1. [Définition 17](#_bookmark34)
      1. [Evénement indésirable 17](#_bookmark35)
      2. [Effet indésirable 17](#_bookmark36)
      3. [Evénement ou effet indésirable grave 17](#_bookmark37)
      4. [Effet indésirable inattendu 17](#_bookmark38)
      5. [Fait nouveau 17](#_bookmark39)
   2. [Description de paramètres d’évaluation de la sécurité et risques liés au](#_bookmark40) [protocole 17](#_bookmark40)
   3. [Evénements indésirables en lien avec le Propofol et le Remifentanil 18](#_bookmark41)
      1. [Evènements indésirables liés au Propofol 18](#_bookmark42)
      2. [Evènements indésirables liés au Remifentanil 19](#_bookmark43)
   4. [Procédures mises en place en vue de l’enregistrement et de la notification](#_bookmark44) [des événements indésirables 20](#_bookmark44)
      1. [Responsabilités de l’investigateur 20](#_bookmark45)
      2. [Responsabilités du promoteur 21](#_bookmark46)

1. [Droit d’accès aux données et documents sources 22](#_bookmark47)
2. [Contrôle et assurance de la qualité 22](#_bookmark48)
3. [Considérations éthiques et légales 23](#_bookmark49)
   1. [Comité de protection des personnes et Autorité compétente 23](#_bookmark50)
      1. [Avis favorable du CPP 23](#_bookmark51)
      2. [Modifications au protocole 23](#_bookmark52)
   2. [Information et consentement 23](#_bookmark53)
4. [Traitement des données et conservation des documents et des données](#_bookmark54) [relatives à la recherche 24](#_bookmark54)
5. [Financement et assurance 25](#_bookmark55)
   1. [Financement 25](#_bookmark56)
   2. [Assurance 25](#_bookmark57)
6. [Publication- Valorisation 25](#_bookmark58)
7. [Liste des annexes 26](#_bookmark59)

## Liste des abréviations

| **ABBREVIATION** | **DEFINITION** |
| --- | --- |
| ANI | Analgesia Nociception Index |
| ANSM | Agence Nationale de Sécurité du Médicament et des produits de santé |
| ARC | Attaché de recherche Clinique |
| ASA | American Society of Anestheology |
| CHRU | Centre Hospitalier Régional Universitaire |
| CNIL | Commission Nationale de l’Informatique et des Libertés |
| CPP | Comité de Protection des Personnes |
| CRF/eCRF | Case Report Form/ electronic Case Report Form |
| EI | Evénement Indésirable |
| EIG | Evénement Indésirable Grave |
| EVA | Echelle Visuelle Analogique |
| FC | Fréquence Cardiaque |
| FDA | Food and Drug Administration |
| PAS | Pression Artérielle Systolique |
| PK / PD | Pharmacokinetics / pharmacodynamic |
| Remi | Remifentanil |
| SNA | Système Nerveux Autonome |
| VFC | Variabilité de la Fréquence Cardiaque |

1. **SYNOPSIS**

| PROMOTEUR | **CHRU de Lille**  Département de la Recherche en Santé  6 rue du Pr Laguesse - 59037 Lille Cedex Tél : 03 20 44 41 45 - Fax : 03 20 44 57 11 |
| --- | --- |
| TITRE | Pilotage automatique de l’administration de remifentanil guidé par l’ANI au cours de l’anesthésie générale au propofol |
| INVESTIGATEUR PRINCIPAL | Dr Mathieu JEANNE |
| NOMBRE DE CENTRES | 1 Centre : Centre de traitement des brulés, Hôpital Roger Salengro, CHRU de Lille |
| TYPE D’ETUDE | Contrôlée, Randomisée, en simple aveugle, Monocentrique de catégorie 1 |
| OBJECTIFS | **Objectif principal**  Déterminer si le dispositif médical ANI-loop permet de diminuer la quantité totale de remifentanil (remi) administrée par rapport à la pratique standard.  **Objectifs secondaires**  Déterminer si l’administration automatisée de remifentanil par le dispositif ANI-loop permet de :   - améliorer la stabilité hémodynamique au cours de l’anesthésie générale au propofol. - augmenter le temps passé avec un ANI compris entre 50 et 70. - diminuer la douleur post-opératoire immédiate. |
| PLAN EXPERIMENTAL | - étude contrôlée randomisée en simple aveugle de catégorie 1 - monocentrique : Centre de Traitement des Brûlés du CHRU de Lille - randomisation réalisée par le pôle promotion médical directement via le CRF électronique - dans le bras *"ANI-loop"*, administration autonome de remifentanil par le dispositif medical ANI-loop (classe IIb) sous la supervision d'un anesthésiste - dans le bras "pratique standard", administration de remifentanil adaptée par l’anesthésiste selon les pratiques habituelles - le moniteur ANI est visible dans les deux bras |
| CRITERES D’EVALUATION | **Critère principal :**  Quantité totale de remi administrée (valeur rapportée au poids et à la durée d'intervention).  **Critères secondaires permettant de répondre aux objectifs secondaires:**   - Proportion de temps passé en réactivité hémodynamique, hypotension ou bradycardie pendant l’intervention. Cette proportion sera égale au cumul des durées de chaque épisode de réactivité, d’hypotension ou de bradycardie rapporté à la durée de l’intervention. - Dose totale d’Ephedrine - ANIi et ANIm : proportion de temps passé pendant |

|  | l’intervention dans les intervalles suivants : <50, [50-70], >70   - indice de mesure de la composante hypnotique (BIS® ou Entropie®) : proportion de temps passé pendant l’intervention dans les intervalles suivants : <40, [40-60], >60 - ANI, fréquence cardiaque, pression artérielle, FetCO2, Pmax (respirateur) 5 min avant le début de l’intervention, 5 min après le début de l’intervention et 20 min après le début de l’intervention. - Douleur évaluée par échelle visuelle analogique (EVA) entre H0 et H2, toutes les 15 min.   Quantité totale de morphine administrée entre fin de chirurgie et H2 post opératoire.   - Quantité totale de kétamine administrée entre fin de chirurgie et H2 post opératoire.   •Fréquence de nausées/vomissements post opératoires évaluée à H2.  Nombre de modifications de débit de remifentanil pendant l’intervention   - Quantité totale de propofol administrée (valeur absolue, valeur rapportée au poids, valeur rapportée au poids et à la durée d'intervention). - Nombre de changements de cible de propofol pendant l’intervention   Dans le bras *"ANI-loop"* uniquement : nombre et durée totale de débrayages du dispositif ANI-loop remi pendant l’intervention, c’est-à-dire le nombre de fois et la durée cumulée où le système de boucle fermé a dû être interrompu et relayé par un pilotage manuel tel que dans le bras pratique standard |
| --- | --- |
| CRITERES D’INCLUSION | - patient pris en charge au Centre de Traitement des Brûlés du CHRU de Lille - chirurgie du brûlé: excision-greffe - âge ≥ 18 ans - consentement éclairé - statut ASA I ou II ou III - anesthésie générale avec intubation et ventilation assistée contrôlée - indice de masse corporelle compris en 17 et 40 kg.m-2 |
| CRITERES DE NON INCLUSION | - femme enceinte ou allaitante (simple interrogatoire) - pace maker (quel que soit le mode de fonctionnement) - rythme cardiaque non sinusal - greffe cardiaque - dysautonomie - diabète sucré avec complication micro ou macro angiopathique - allergie ou intolérance prévisible à un médicament utilisé pour l’étude - mesures hémodynamiques “baseline” considérées comme “réactivité”, hypotension ou bradycardie - FCbl>120 min^-1^ ou PASbl>160 mmHg - non assuré social - personnes privées de liberté, en situation d’urgence, ou personnes incapables de consentir et ne bénéficiant pas d’un régime de protection juridique (tutelle/curatelle) - patient inconscient ou sédaté avant induction |
| NOMBRE DE PARTICIPANTS | L’objectif de l’étude est d’évaluer si le dispositif médical ANI-loop permet de diminuer la quantité totale de remifentanil administré par rapport à la pratique standard. Sur la base des données de la littérature [Leal PC et al.] et de la pratique habituelle du |

|  | service, la quantité totale de remifentanil dans le groupe contrôle peut-être estimée à 0.30 ± 0.075 (moyenne ± écart-type). Pour mettre en évidence une diminution de 20% dans le bras *"ANI- loop"* (soit une quantité moyenne de remifentanil de 0.24 dans le groupe expérimental), avec un risque de première espèce de 5%, une puissance de 80%, il est nécessaire d’inclure 26 patients par groupe, soit 52 patients au total. |
| --- | --- |
| STRATEGIE D’ANALYSE STATISTIQUE | Les analyses statistiques seront réalisées à l’aide du logiciel SAS (version 9.4 ou supérieure) et conduite à la plateforme d’aide méthodologique du CHRU de Lille sous la responsabilité du Pr A. Duhamel. Pour répondre à l’objectif principal, la quantité totale de remifentanil administrée (valeur rapportée au poids et à la durée d'intervention) sera comparée entre les deux bras de l’étude par un test t de Student ; la taille de l’effet sera calculée (différence moyenne standardisée) avec un intervalle de confiance à 95%. En cas d’écart à la normalité de la distribution du critère principal, le test U de Mann-Whitney sera utilisé. |
| PROCEDURE D’INVESTIGATION SPECIFIQUE A L’ETUDE ET DIFFERENCES PAR RAPPORT A LA PRISE EN CHARGE HABITUELLE | - bras "pratique standard" : la prise en charge est identique à celle d’une anesthésie générale totale intra veineuse - bras *"ANI-loop"* : la prise en charge ne diffère de la pratique standard que pour l’administration de remi, qui est administrée par l'intermédiaire du dispositif ANI-loop. |
| EVALUATION DES BENEFICES ET DES RISQUES LIES À LA RECHERCHE | BENEFICE  L’utilisation du dispositif devrait résulter en une hémodynamique plus stable et donc en une meilleure qualité d’anesthésie, ainsi que potentiellement en une administration totale plus faible de remi.  RISQUE   - bras "pratique standard" : le risque est identique à celui de toute anesthésie générale - bras *"ANI-loop"*: en cas de défaillance du dispositif ANI- loop, l’anesthésiste peut le désengager à tout moment et enchaîner la prise en charge anesthésique selon la "pratique standard" sans délai. Le risque est donc comparable au bras "pratique standard" |
| PERIODE D’EXCLUSION | La participation simultanée à une autre étude est interdite parce qu’elle ferait courir le risque d’interactions avec les réponses du système nerveux autonome. Cette interdiction est limitée à la durée de l’étude, c'est-à-dire à la phase péri opératoire. |
| JUSTIFICATION DE LA CONSTITUTION OU NON D’UN COMITE DE SURVEILLANCE | Non, au vu du risque similaire dans le bras *"ANI-loop"* et dans le bras "pratique standard", la constitution d'un comité de surveillance ne parait pas justifiée. |
| DUREE DE L’ETUDE | - Durée de la période d’inclusion : 12 mois - Durée de participation à l’étude pour un sujet : de l’arrivé au bloc jusqu’à H2 post opératoire - Durée de la recherche : 15 mois - Durée d’analyse des données : 3 mois |

## Rationnel : Justification scientifique et description générale de la recherche

La prise en charge de la douleur en milieu hospitalier est une démarche qui s’inscrit dans le cadre de l’amélioration de la qualité des soins. La difficulté pour les soignants réside dans l’appréciation du niveau de douleur afin d’adapter la prescription et limiter les risques d'effets indésirables de ces médicaments^1^. L’anesthésie générale combine des médicaments hypnotiques et analgésiques afin de permettre la réalisation d’actes chirurgicaux. La surveillance des données cliniques et paracliniques telles que le pouls et la pression artérielle utilisés pour la surveillance du patient sous anesthésie générale ne permettent pas d’anticiper précisément les besoins en antalgiques des patients. Par ailleurs, les médicaments puissants utilisés exposent au risque constant de surdosage, et imposent de rechercher les doses minimales nécessaires pour la réalisation du geste chirurgical. A contrario, une analgésie insuffisante entraînerait un retentissement cardiovasculaire (en particulier une augmentation de la fréquence cardiaque ou de la pression artérielle) qui serait néfaste pour la conduite de l’acte chirurgical (majoration du saignement) et pourrait entraîner des risques d’infarctus chez le sujet coronarien, ainsi que des complications lors de la phase de réveil (douleurs, anxiété). La qualité de l’anesthésie repose sur l’utilisation optimale des produits anesthésiques qui évitent au patient, même inconscient, de ressentir la douleur liée à l’acte chirurgical. La recherche de la dose minimale efficace en antalgiques morphiniques constitue également un objectif majeur de l'anesthésie moderne afin de limiter le risque d'hyperalgésie post-opératoire^2,3^. On peut attendre de l'optimisation de l'administration des antalgiques une amélioration des conditions de réveil et une diminution du risque de complications péri-opératoires, ce qui pourrait contribuer à l’évolution des pratiques cliniques en faveur d’une diminution de la durée d’hospitalisation.

Certaines études ont clairement démontré que les processus de régulation du système cardiovasculaire sont étroitement liés à la perception de la douleur. L’analyse de la variabilité de la fréquence cardiaque (VFC) est une méthode connue et non invasive pour la mesure de la régulation du système cardiovasculaire par le système nerveux autonome (SNA). Cette analyse est basée sur le principe selon lequel les fluctuations du rythme cardiaque sont le reflet de l’activité des systèmes sympathique et parasympathique, principales composantes du SNA^4^. Des études ont montré que les fluctuations de la fréquence cardiaque au-delà de 0.15 Hz sont exclusivement dues à l’influence du système parasympathique. Chez l’adulte, les phénomènes douloureux, de peur ou d’anxiété sont accompagnés d’une diminution de la VFC dans les hautes fréquences (>0.15 Hz) indiquant une diminution du tonus parasympathique au cours de stimuli ou d’émotions déplaisants. Pendant l’intervention chirurgicale, l’analyse de la VFC est corrélée à la balance entre nociception et niveau d’analgésie. Chez l’enfant, une diminution significative de la puissance spectrale dans les hautes fréquences a été observée au cours d’actes chirurgicaux douloureux suggérant une diminution de l’influence parasympathique au cours du stimulus nociceptif. L'équipe lilloise, qui regroupe des cliniciens et des scientifiques associés au Centre d’Investigation Clinique – Innovation Technologique (INSERM CIC-IT 1403) du CHRU de Lille, travaille depuis plusieurs années sur l'analyse de la VFC. Ces différents travaux ont aboutis à la création d’un l’indice original de VFC lié à la balance analgésie – nociception : l’ANI (Analgesia Nociception Index). Cette technologie à fait l’objet de plusieurs dépôts de brevets. Cette technologie est désormais disponible par l’intermédiaire du dispositif Physiodoloris® (Mdoloris Medical Systems®, Loos, France). Ce dispositif marqué CE médical est actuellement utilisé en routine clinique dans de nombreux hôpitaux^5-9^.

De récentes études ont montré que l’utilisation du dispositif Physiodoloris® pour adapter la composante analgésique de l’anesthésie générale permettait de limiter la survenue et la durée des épisodes de réactivité délétères pour le patient tout en réduisant de manière significative la consommation per opératoire en morphinique. La plupart de ces études avaient pour but de réguler l’administration de remifentanil en changeant manuellement le débit des pousses seringues en fonction des valeurs affichées de l’ANI. Grace à notre expérience de l’utilisation de l’ANI au cours de l’anesthésie générale, nous avons établi différentes règles de décision basées sur l’analyse de l’ANI et de la pression artérielle, permettant de guider l’anesthésiste pour la prise en charge de l’analgésie per opératoire.

Notre dernière innovation concerne la réalisation d’un dispositif médical qui permet l'automatisation de l'administration du remifentanil selon un algorithme pré-établi. Cette innovation a fait l’objet de deux dépôts de brevet. Le dispositif médical ANI-loop se présente sous la forme d’un contrôleur implémenté sur un mico-ordinateur, relié au scope multiparamétrique utilisé pour la surveillance de l’anesthésie, ainsi qu'au moniteur Physioldoloris® et à un pousse-seringue électrique (Annexe I).

Nous proposons de réaliser une étude randomisée contrôlée afin de déterminer si l'utilisation du dispositif medical ANI-loop au cours de l’anesthésie générale au propofol permet de diminuer la dose totale de remifentanil administrée.

## Objectif de la recherche

### Objectif principal

Déterminer si le dispositif médical ANI-loop permet de diminuer la quantité totale de remifentanil (remi) administrée par rapport à la pratique standard.

### Objectifs secondaires et les hypothèses générées

Déterminer si l’administration automatisée de remifentanil par le dispositif ANI-loop permet de :

- améliorer la stabilité hémodynamique au cours de l’anesthésie générale au propofol.
- augmenter le temps passé avec un ANI compris entre 50 et 70.
- diminuer la douleur post-opératoire immédiate.

Dans le bras *"ANI-loop"* uniquement : déterminer si ANI-loop permet une administration complètement automatisée (i.e. sans qu'aucun débrayage du dispositif ne soit nécessaire).

## Conception de la recherche

### Plan expérimentale

- étude contrôlée randomisée en simple aveugle de catégorie 1 versus pratique standard
- monocentrique : Centre de Traitement des Brûlés du CHRU de Lille randomisation réalisée par le pôle promotion médical directement via le CRF électronique
- dans le bras *"ANI-loop"*, administration autonome de remifentanil par le dispositif médical ANI- loop sous la supervision d'un anesthésiste
- dans le bras "pratique standard", administration de remifentanil adaptée par l’anesthésiste selon les pratiques habituelles
- le moniteur ANI est visible dans les deux bras

### Définition de la population étudiée

La mesure ANI fournie par le moniteur PhyioDoloris n’est pas interprétable dans plusieurs situations. L’ensemble des critères concernés sont pris en compte dans les critères de non inclusion ou d’exclusion ;

- arythmie
  - Critère d’exclusion
- absence de respiration (ex : apnée due à l’intubation)
  - Non concerné ; le dispositif ANI-loop n’est pas utilisé en mode automatique lorsque le patient n’est pas intubé et ventilé mécaniquement.
- fréquence respiratoire inférieure à 9 cycles/min
  - Non concerné ; le protocole prévoit une fréquence respiratoire à 12 cycles minutes
- volume courant variable sur la durée de mesure, soit 64 secondes
  - Non concerné ; pour ce genre d’anesthésie, les adaptions de volumes courant se font habituellement au maximum 1 à 2 fois par heure. Les changements importants de volume courant sur une période de 64 secondes ne sont pas pratiqués dans ce contexte clinique.
- respiration irrégulière (quand le sujet parle, rit ou tousse)
  - Non concerné ; le dispositif ANI-loop n’est pas utilisé en mode automatique lorsque le patient n’est pas intubé et ventilé mécaniquement.
- pace maker (certains types)
  - Critère de non inclusion
- greffe cardiaque
  - Critère de non inclusion
- Utilisation de drogues ayant un effet sur l’activité cardiaque sinusale (Atropine…)
  - Critères d’excusions ; injections d’atropine (parasympatholytique) et de propranolol (sympatholytique)

###### Critères d’inclusion

- patient pris en charge au Centre de Traitement des Brûlés du CHRU de Lille
- chirurgie du brûlé: excision-greffe
- âge ≥ 18 ans
- consentement éclairé
- statut ASA I ou II ou III
- anesthésie générale avec intubation et ventilation assistée contrôlée
- indice de masse corporelle compris en 17 et 40 kg.m-2

###### Critères de non-inclusion

- femme enceinte ou allaitante (simple interrogatoire)
- pace maker (quel que soit le mode de fonctionnement)
- greffe cardiaque
- rythme cardiaque non sinusal
- dysautonomie
- diabète sucré avec complication micro ou macro angiopathique
- allergie ou intolérance prévisible à un médicament utilisé pour l’étude
- mesures hémodynamiques “baseline” considérées comme hypotension ou bradycardie
- FCbl>120 min^-1^ ou PASbl>160 mmHg
- non assuré social
- personnes privées de liberté, en situation d’urgence, ou personnes incapables de consentir et ne bénéficiant pas d’un régime de protection juridique (tutelle/curatelle)
- patient inconscient ou sédaté avant induction

NB : Il n’y a pas de test de grossesse prévu pour la vérification de ce critère, il s’agira d’un simple interrogatoire

###### Critère d’exclusion

- Arythmie
- extrasystolie importante (plus de deux extrasystoles par minute sans interruption pendant la chirurgie)
- Injection per-opératoire d’atropine
- Injection per-opératoire de propranolol.

En cas d'exclusion d'un patient, son inclusion est réputée nulle ; le centre investigateur procédera alors à une inclusion supplémentaire en remplacement. Aucun suivi du patient exclu n’est justifié par la participation à l’étude.

### Critères d’évaluation

###### Définitions

- *les valeurs “baseline”* pour la fréquence cardiaque (FCbl) et la pression artérielle systolique (PASbl) sont la moyenne de 3 mesures réalisées avant le début de l’anesthésie
- *réactivité hémodynamique* : FC > 120% de FCbl ou PAS > 120% de PASbl
- *hypotension* : PAS <75 mmHg
- *bradycardie* : FC < 40 min-1
- *intervention* : débute avec la préparation cutanée, inclut la chirurgie et termine avec la fin du pansement

###### critère principal, permettant de répondre à l’objectif principal

Quantité totale de remi administrée (valeur rapportée au poids et à la durée d'intervention).

###### critères secondaires permettant de répondre aux objectifs secondaires:

NB : plusieurs critères sont évalués après le réveil du patient, entre l'extubation (H0) et H2 120 min après extubation. Le patient ne nécessite pas d'être en SSPI pour cette évaluation, il peut être dans sa chambre d'hospitalisation.

- Proportion de temps passé en réactivité hémodynamique, hypotension ou bradycardie pendant l’intervention. Cette proportion sera égale au cumul des durées de chaque épisode de réactivité, d’hypotension ou de bradycardie rapporté à la durée de l’intervention Dose totale d’Ephedrine.
- ANIi et ANIm : proportion de temps passé pendant l’intervention dans les intervalles suivants :

<50, [50-70], >70

- indice de mesure de la composante hypnotique (BIS® ou Entropie®) : proportion de temps passé pendant l’intervention dans les intervalles suivants : <40, [40-60], >60
- ANI, fréquence cardiaque, pression artérielle, FetCO2, Pmax (respirateur) 5 min avant le début de l’intervention, 5 min après le début de l’intervention et 20 min après le début de l’intervention.
- Douleur évaluée par échelle visuelle analogique (EVA) entre H0 et H2, toutes les 15 min.
- Quantité totale de morphine administrée entre fin de chirurgie et H2 post opératoire.
- Quantité totale de kétamine administrée entre fin de chirurgie et H2 post opératoire.
- Fréquence de nausées/vomissements post opératoires évaluée à H2.
- Nombre de modifications de débit de remifentanil pendant l’intervention.
- Quantité totale de propofol administrée (valeur absolue, valeur rapportée au poids, valeur rapportée au poids et à la durée d'intervention).
- Nombre de changements de cible de propofol pendant l’intervention.

Dans le bras *"ANI-loop"*uniquement : nombre et durée totale de débrayages du dispositif ANI-loop remi pendant l’intervention, c’est-à-dire le nombre de fois et la durée cumulée où le système de boucle fermé a dû être interrompu et relayé par un pilotage manuel tel que dans le bras pratique standard

### Calcul du nombre de sujets

L’objectif de l’étude est d’évaluer si le dispositif medical ANI-loop permet de diminuer la quantité totale de remifentanil (remi) administrée par rapport à la pratique standard. Sur la base des données de la littérature [Leal PC et al.] et de la pratique habituelle du service, la quantité totale de remifentanil dans le groupe contrôle peut-être estimée à 0.30 ± 0.075 (moyenne ± écart-type). Pour mettre en évidence une diminution de 20% dans le bras *"ANI-loop"* (soit une quantité moyenne de remifentanil de 0.24 dans le groupe expérimental), avec un risque de première espèce de 5%, une puissance de 80%, il est nécessaire d’inclure 26 patients par groupe, soit 52 patients au total.

Reference:Leal PC, Sakata RK, Salomão R, Sadatsune EJ, Issy AM. Braz J Anesthesiol. 2013 Mar- Apr;63(2):178-82.

### Méthode et stratégie d’analyse

Les analyses statistiques seront réalisées à l’aide du logiciel SAS (version 9.4 ou supérieure) et conduite à la plateforme d’aide méthodologique du CHRU de Lille sous la responsabilité du Pr A. Duhamel. Tous les tests statistiques seront bilatéraux avec un risque de première espèce de 5%. Il n’est pas prévu de faire des analyses intermédiaires. Un plan d’analyse statistique détaillé sera rédigé et validé avant le gel de la base de données.

Les caractéristiques des patients à l’inclusion seront décrites pour chacun des deux bras de l’étude. Les variables qualitatives seront décrites par les effectifs et pourcentages. Les variables quantitatives seront décrites par la moyenne et l’écart type en cas de distribution gaussienne, ou par la médiane et l’interquartile (i.e. 25ième et 75ième percentiles) dans le cas contraire. La normalité des distributions sera testée par un test de Shapiro-Wilk et vérifiée graphiquement par des histogrammes.

Objectif principal : La quantité totale de remifentanil administrée (valeur rapportée au poids et à la durée d'intervention) sera comparée entre les deux bras de l’étude par un test t de Student ; la taille de l’effet sera calculée (différence moyenne standardisée) avec un intervalle de confiance à 95%. En cas d’écart à la normalité de la distribution du critère principal, le test U de Mann-Whitney sera utilisé.

Objectifs secondaires : Les critères secondaires qualitatifs seront comparés entre les deux bras de l’étude à l’aide d’un test du Chi-deux ou par un test exact de Fisher (lorsque les conditions de validité du test du Chi-deux ne sont pas vérifiées). Les critères secondaires quantitatifs seront comparés entre les deux bras de l’étude par un test t de Student ou par un test U de Mann-Whitney en cas d’écart à la normalité de la distribution du critère. Les critères secondaires quantitatifs mesurés à différent temps seront comparés entre les deux bras de l’étude à l’aide d’un modèle linéaire mixte. Ce modèle permet de prendre en compte la corrélation entre les mesures répétées chez un même patient, et l’existence éventuelle de données manquantes. Le choix du modèle de corrélation reposera sur le critère d’AIC. La validité du modèle sera étudiée par l’analyse des résidus. En cas d’écart important à la normalité sans transformation évidente du critère, les critères seront comparés à chaque temps entre les groupes par des tests U de Mann-Whitney. Enfin, les critères secondaires relevés uniquement dans le groupe

« *ANI-loop* » seront décrits par les statistiques descriptives usuelles.

## Logistique de l’étude

### Equipes participantes et expérience de l’équipe dans le domaine

##### Pôle d’Anesthésie Réanimation (Professeur Benoît TAVERNIER), CHRU Lille :

La clinique d'anesthésie-réanimation R. Salengro est une structure regroupant et coordonnant l'ensemble des activités d'anesthésie-réanimation de l'hôpital Roger Salengro. Partie prenante du pôle d'anesthésie-réanimation du CHRU et du Département Universitaire d'anesthésie-réanimation et de médecine d'urgence de la Faculté de Médecine, les missions prioritaires du pôle sont le soin, l'innovation, l'enseignement et la recherche en anesthésie-réanimation.

La clinique R. Salengro est composé de six secteurs d'activité d'anesthésie-réanimation (urgences, orthopédie-traumatologie, "spécialités chirurgicales", neurochirurgie - neuroradiologie, réanimation neurochirurgicale, centre de traitement des brûlés). L'ensemble de la structure compte 46 anesthésistes réanimateurs (hospitalo-universitaires et hospitaliers), 19 internes, pour des activités d'anesthésie réanimation et médecine péri opératoire organisées autour de 40 salles opératoires ou

interventionnelles, 36 lits de SSPI, et près de 400 lits d'hospitalisation chirurgicale, dont 18 lits de réanimation chirurgicale et 26 de soins intensifs chirurgicaux.

Membres de l’équipe participant à la recherche : Dr Mathieu JEANNE (PH), Dr Michel Delecroix (PH), Dr Arnaud ALLUIN (CCU-AH).

##### INSERM CIC-IT 1403, CHRU Lille:

Labellisé en janvier 2008, par l’INSERM et la DHOS, le Centre d’Investigation Clinique et d’Innovations Technologiques (CIC-IT) est une unité fonctionnelle du CHRU de Lille. Il possède une compétence spécifique nationale sur la thématique « Biocapteurs et e-Santé » concernant l’innovation et les usages. Il constitue une structure entièrement dédiée à la réalisation de projets de recherches cliniques impliquant des innovations technologiques, institutionnels ou industriels, en collaboration avec le CIC Plurithématique. Le CIC-IT de Lille réunit deux équipes spécialisées dans le domaine : - l’équipe « Biocapteurs et Instrumentation » spécialisée dans le développement de technologies nouvelles dans le domaine de l’acquisition et du traitement des signaux physiologiques, - le laboratoire EVALAB, spécialisé dans l’Utilisabilité des applications technologiques en Médecine.

Le CIC-IT bénéficie des infrastructures du CHRU de LILLE, il est installé à la Maison Régionale de la Recherche Clinique, sur la campus du CHRU de Lille.

Membres de l’équipe participant à la recherche : Dr Julien DE JONCKHEERE (chargé de mission de recherche), Mathilde FLOCTEIL (attachée de recherche clinique), Dr Mathieu JEANNE (PH).

### Déroulement pratique de la recherche

###### Consentement éclairé

Il sera obtenu après information lors de la consultation pré anesthésique ou lors de la visite pré anesthésique ultime, par un des investigateurs ou des investigateurs. Le patient recevra le formulaire d’information et devra signer le formulaire de consentement en trois exemplaires, après un délai de réflexion raisonnable.

###### Dispositifs utilisés pour l'anesthésie

L’anesthésie générale est induite et entretenue par propofol et remifentanil.

- Le propofol est administré dans les deux bras par un système d’anesthésie intra veineuse à objectif de concentration selon un modèle pK/pD: base Primea Orchestra® (Fresenius) utilisée en routine dans le service.
- Le remifentanil est administré
  - dans le bras "*pratique standard*", par un système d’anesthésie intra veineuse à objectif de concentration selon un modèle pK/pD : base Primea Orchestra® (Fresenius) utilisée en routine dans le service. Les cibles autorisées selon les pratiques habituellement constatées vont de 0.0 à 10.0 ng.ml^-1^ pendant l’anesthésie.
  - Dans le bras "*ANI-loop*", le remifentanil est administré automatiquement par le dispositif medical ANI-loop.

###### Déroulement de l'anesthésie

*Accueil du patient au bloc opératoire*

Installation des moyens de surveillance habituels

ECG, pléthysmographie, pression artérielle non invasive (PNI), Entropie, ANI.

Recueil des constantes hémodynamiques habituelles et calcul des seuils FC_bl_ et PAS_bl_utilisés en per anesthésique

Randomisation directement via le CRF électronique et attribution au bras *"pratique standard"* ou *"ANI- loop"*.

Le moniteur Physiodoloris® (indice ANI) demeure visible dans les deux bras de l'étude.

*Induction de l'anesthésie*

Induction anesthésique similaire dans les deux groupes : midazolam 0,08 mg.kg^-1^ IVD

cible de remifentanil fixée à 8.0 ng.ml^-1^

cible de propofol fixée à 5.0 µg.ml^-1^ puis augmentée par paliers de 1 µg.ml^-1^ jusqu’à la perte de conscience.

Intubation trachéale, éventuellement après curarisation

cibles de propofol et remifentanil baissées resp. à 3 µg.ml^-1^ et 3 ng.ml^-1^

Ventilation Assistée Contrôlée (VAC) : volume courant de 8 ml.kg^-1^ de poids idéal, fréq. ventilatoire : 12 cycles.min^-1^

Le volume courant sera adapté pour maintenir la FetCO2 dans l'intervalle [30 - 35 mmHg]

*Entretien de l’anesthésie pendant l'intervention*

- cible de propofol adaptée dans les deux bras pour maintenir l'indice de mesure de la composante hypnotique (BIS® ou Entropie®) dans l’intervalle [40-60]. Pendant *l'intervention*, les modifications de cible de propofol seront faites 1 minute après que le dernier changement de cible est devenu effectif, et suivront les indications du tableau 1. Ceci correspond à la prise en charge habituelle au bloc du Centre de Traitement des Brulés.

|  |  |  |
| --- | --- | --- |
| composante  hypnotique | cible propofol |  |
| >80 | + 2.0 |  |
| >60 | + 1.0 |  |
| <40 | - 0.5 |  |
| <25 | - 1.0 |  |
| **Tableau 1 : adaptation des cibles de propofol dans les deux bras** | | |

|  |  |  |
| --- | --- | --- |
| ANI | cible remi |  |
| <35 | + 2.0 |  |
| <50 | + 1.0 |  |
| >70 | - 0.5 |  |
| >90 | - 1.0 |  |
| **Tableau 2 : adaptation des cibles de remi dans le bras "pratique standard"** | | |

- cible de remifentanil
  - **dans le bras *pratique standard***: augmentée à 4 ng.ml-1 au début de *l'intervention*, puis adaptée afin de maintenir l’ANI dans l’intervalle [50-70] selon les indications du tableau 2. Les changements de cible seront faits 1 minute après que le dernier changement de cible est devenu effectif. Ceci correspond à la prise en charge habituelle au Centre de Traitement des Brulés.
  - **dans le bras "*ANI-loop"***, le mode automatique du dispositif ANI-loop est enclenché au début de *l'intervention*. Le débit de remifentanil est régulé automatiquement par l'ANI- loop. L’anesthésiste a la possibilité de passer en mode manuel à tout moment en cas de régulation inadaptée (débrayage du dispositif). Le dispositif est stoppé à la fin de *l'intervention.*

##### Analgésie préemptive

- infiltration locale de la zone de prélèvement cutané avec naropeine 2 mg.ml-1 au cours de

*l'intervention*

- paracétamol 1g IVL en fin *d'intervention*
- sufentanil 0,12 µg.kg-1 (max 10 µg) IVL en *fin d'intervention*

##### Prévention des nausées/vomissements post opératoires

- ondansetron 4 mg IVL

##### Analgésie post opératoire

- titration par morphine selon Echelle Visuelle Analogique (EVA), bolus de 3mg toutes les 3 min (max 12mg)
- kétamine 20 mg IVL en cas de douleur persistante avec EVA ≥ 40

### Durée

Le promoteur se réserve le droit d’interrompre l’essai en raison d’un défaut d’inclusion. L’étude pourra être arrêtée par décision conjointe de l’autorité compétente, du promoteur ou de l’investigateur principal.

- durée de la période d’inclusion : 12 mois
- durée de participation à l’étude pour un sujet : de l’arrivé au bloc jusqu’à H2 post opératoire
- durée de la recherche : 15 mois
- durée d’analyse des données : 3 mois

### Critères d’arrêt de participation à l’étude

Chaque sujet pourra sortir de l’étude par décision de l’autorité administrative compétente, du promoteur et de l’investigateur principal mais aussi par décision d’un investigateur ou par décision de l’intéressé lui-même conformément à la réglementation et comme il est mentionné dans le formulaire de recueil du consentement.

- la présence d’une extrasystolie importante (plus de deux extrasystoles par minute sans interruption) constitue un critère d’arrêt de participation d’un patient à l’étude, parce que les mesures de l’ANI ne sont pas possibles dans ces conditions.
- dans ce cas, l’inclusion du patient serait réputée nulle ; le centre investigateur procéderait alors à une inclusion supplémentaire en remplacement.
- aucun suivi du patient exclu n’est justifié par la participation à l’étude.

### Interdiction de participation simultanée – Période d’exclusion

La participation simultanée à une autre étude est interdite parce qu’elle ferait courir le risque d’interactions avec les réponses du système nerveux autonome. Cette interdiction est limitée à la durée de participation du sujet à l’étude

### Bénéfices, risques et contraintes de l’étude

###### BENEFICE

L’utilisation du dispositif devrait résulter en une hémodynamique plus stable et donc en une meilleure qualité d’anesthésie, ainsi que potentiellement en une administration totale plus faible de remi.

###### BENEFICE COLLECTIF

Une anesthésie de meilleure qualité bénéficierait probablement à long terme à l'ensemble de la qualité des soins péri-opératoires.

###### RISQUES

- bras "pratique standard" : le risque est identique à celui de toute anesthésie générale
- bras *"ANI-loop"* : en cas de défaillance du dispositif ANI-loop, l’anesthésiste peut le désengager à tout moment et enchaîner la prise en charge anesthésique selon la "pratique standard" sans délai. Le risque est donc comparable au bras "pratique standard"

###### Comité de Surveillance

- au vu du risque similaire dans le bras *"ANI-loop"* et dans le bras "pratique standard", la constitution d'un comité de surveillance ne parait pas justifiée.

## Dispositif médical

Annexes II.

## Evaluation de la sécurité

### Définition

###### Evénement indésirable

Toute manifestation nocive survenant chez une personne qui se prête à une recherche impliquant la personne humaine que cette manifestation soit liée ou non à la recherche ou au produit sur lequel porte cette recherche.

###### Effet indésirable

Tout événement indésirable lié à la recherche ou au produit sur lequel porte cette recherche.

###### Evénement ou effet indésirable grave

Tout événement ou effet indésirable qui :

- entraîne la mort,
- met en danger la vie de la personne qui se prête à la recherche,
- nécessite une hospitalisation ou la prolongation d’une hospitalisation,
- provoque une incapacité ou un handicap important ou durable,
- ou bien se traduit par une anomalie ou une malformation congénitale, et s’agissant du médicament, quelle que soit la dose administrée.
- est jugé comme grave sur le plan médical par l’investigateur.

Certaines circonstances nécessitant une hospitalisation ne relèvent pas du critère de gravité

« hospitalisation ou la prolongation d’une l’hospitalisation » comme :

- admission pour des raisons sociales ou administratives
- hospitalisation prédéfinie par le protocole
- hospitalisation pour traitement médical ou chirurgical programmé avant la recherche
- passage en hôpital de jour

###### Effet indésirable inattendu

Tout effet indésirable dont la nature, la sévérité ou l’évolution ne concorde pas avec les informations relatives aux produits, actes pratiqués et méthodes utilisées au cours de la recherche.

###### Fait nouveau

Toute nouvelle donnée pouvant conduire à une réévaluation du rapport des bénéfices et des risques de la recherche ou du produit objet de la recherche, à des modifications dans l'utilisation de ce produit, dans la conduite de la recherche, ou des documents relatifs à la recherche, ou à suspendre ou interrompre ou modifier le protocole de la recherche ou des recherches similaires.

### Description de paramètres d’évaluation de la sécurité et risques liés au protocole

- bras "pratique standard" : le risque est identique à celui de toute anesthésie générale
- bras *"ANI-loop"*: en cas de défaillance du dispositif ANI-loop, l’anesthésiste peut le désengager à tout moment et enchaîner la prise en charge anesthésique selon la "pratique standard" sans délai. Le risque est donc comparable au bras "pratique standard"

### Evénements indésirables en lien avec le Propofol et le Remifentanil

###### Evènements indésirables liés au Propofol

L'induction et le maintien de l'anesthésie ou de la sédation avec le propofol sont en général aisés avec des signes minimes d'excitation. Les effets indésirables les plus fréquemment rapportés pour le propofol sont les effets indésirables pharmacologiquement prévisibles pour un agent anesthésique/sédatif, comme l'hypotension. La nature, la gravité et l'incidence des événements indésirables observés chez les patients recevant du propofol peuvent être liées à l'état des patients et aux procédures opératoires ou thérapeutiques mises en œuvre :

##### Tableau des réactions médicamenteuses indésirables

| **Classe de systèmes d'organes** | **Fréquence** | **Effets indésirables** |
| --- | --- | --- |
| **Affections du système immunitaire :** | *Très rare*  (<1/10 000) | Anaphylaxie - peut comporter angiœdème, bronchospasme, érythème  et hypotension |
| **Troubles du métabolisme et de la nutrition:** | *Fréquence indéterminée*  (9) | Acidose métabolique ^(5)^, hyperkaliémie  ^(5)^, hyperlipidémie ^(5)^ |
| **Affections psychiatriques :** | *Fréquence indéterminée*  (9) | Humeur euphorique, abus de médicaments et pharmacodépendance  (8) |
| **Affections du système nerveux :** | *Fréquent*  (>1/100, <1/10) | Céphalées pendant la phase de réveil |
|  | *Rare*  (>1/10 000, <1/1 000) | Mouvements épileptiformes, dont convulsions et opisthotonos pendant l'induction, le maintien et le réveil |
|  | *Très rare*  (<1/10 000) | Inconscience postopératoire |
|  | *Fréquence indéterminée*  (9) | Mouvements involontaires |
| **Affections cardiaques :** | *Fréquent*  (>1/100, <1/10) | Bradycardie ^(1)^ |
|  | *Très rare*  (<1/10 000) | Œdème pulmonaire |
|  | *Fréquence indéterminée*  (9) | Arythmie cardiaque ^(5)^, insuffisance cardiaque ^(5)^, ^(7)^ |
| **Affections vasculaires :** | *Fréquent*  (>1/100, <1/10) | Hypotension ^(2)^ |
|  | *Peu fréquent*  (>1/1 000, <1/100) | Thrombose et phlébite au site d'injection |
| **Affections respiratoires, thoraciques et médiastinales :** | *Fréquent*  (>1/100, <1/10) | Apnée transitoire pendant l'induction |
|  | *Fréquence indéterminée*  (9) | Dépression respiratoire (dose - dépendante) |
| **Affections gastro-intestinales:** | *Fréquent*  (>1/100, <1/10) | Nausées et vomissements pendant la phase de réveil |
|  | *Très rare*  (<1/10 000) | Pancréatite |
| **Affections hépatobiliaires :** | *Fréquence indéterminée*  (9) | Hépatomégalie ^(5)^ |
| **Affections musculo- squelettiques et systémiques:** | *Fréquence indéterminée*  (9) | Rhabdomyolyse ^(3)^, ^(5)^ |
| **Affections du rein et des voies urinaires :** | *Très rare*  (<1/10 000) | Décoloration de l'urine après administration prolongée |
|  | *Fréquence indéterminée*  (9) | Insuffisance rénale ^(5)^ |

| **Classe de systèmes d'organes** | **Fréquence** | **Effets indésirables** |
| --- | --- | --- |
| **Affections des organes de reproduction et du sein :** | *Très rare*  (<1/10 000) | Désinhibition sexuelle |
| **Troubles généraux et anomalies au site d'administration :** | *Très fréquent*  (>1/10) | Douleur locale à l'induction ^(4)^ |
|  | *Très rare*  (<1/10 000) | Nécrose tissulaire ^(10)^ 'suite à une administration extravasculaire accidentelle |
|  | *Fréquence indéterminée*  (9) | Douleur locale, œdème, suite à une  administration extravasculaire accidentelle |
| **Investigations :** | *Fréquence indéterminée*  (9) | ECG de type Brugada ^(5)^, ^(6)^ |
| **Lésions, intoxications et complications liées à**  **l'intervention :** | *Très rare*  (<1/10 000) | Fièvre postopératoire |

^(1)^ Les bradycardies graves sont rares. Il a été reçu des cas isolés d'évolution vers une asystolie.

^(2)^ L'hypotension peut occasionnellement nécessiter l'emploi de liquides intraveineux et la réduction de la vitesse d'administration du propofol.

^(3)^ De très rares cas de rhabdomyolyse ont été rapportés lorsque le propofol a été administré à des doses supérieures à 4 mg/kg/h pour une sédation en unité de soins intensifs.

^(4)^ La douleur locale peut être minimisée en utilisant de plus grosses veines de l'avant-bras et de la fosse antécubitale. Avec PROPOFOL LIPURO 10 mg/ml la douleur locale peut aussi être minimisée par la co-administration de lidocaïne.

^(5)^ Des combinaisons de ces événements, appelées « syndrome de la perfusion du propofol », peuvent être observées chez les patients gravement malades qui présentent souvent des facteurs de risque multiples pour le développement d'événements, voir rubrique 4.4.

^(6)^ ECG de type Brugada - sus-décalage du segment ST avec aspect en dôme et onde T négative à l'ECG.

^(7)^ Insuffisance cardiaque rapidement évolutive (avec une issue fatale dans certains cas) chez les adultes. Dans de tels cas, l'insuffisance cardiaque ne répond en général pas au traitement inotrope de soutien.

^(8)^ Abus médicamenteux et dépendance médicamenteuse au propofol, le plus souvent par des professionnels de la santé.

^(9)^ Fréquence indéterminée (ne peut être estimée sur la base des données disponibles).

^(10)^ Une nécrose a été rapportée lorsque les tissus ont été endommagés.

###### Evènements indésirables liés au Remifentanil

Les effets indésirables les plus fréquemment observés avec le rémifentanil découlent directement de la pharmacologie des agonistes morphiniques. Ces effets indésirables disparaissent dans les minutes suivant l'arrêt ou la diminution du débit d'administration du rémifentanil.

Les fréquences des effets indésirables ci-dessous sont définies comme : très fréquents (≥ 1/10), fréquents (≥ 1 /100 et < 1/10), peu fréquents (≥ 1/1 000 et < 1/100), rares (≥ 1/10 000 et < 1/1 000) et très rares (< 1/10 000).

| **Affections du système immunitaire** | |
| --- | --- |
| Rares | Des réactions allergiques incluant des réactions anaphylactiques ont été rapportées chez des patients ayant reçu du rémifentanil en association avec un ou plusieurs agents anesthésiques. |
| **Affections du système nerveux** | |
| Très fréquents | Rigidité des muscles du squelette |
| Rares | Sédation (durant la phase de réveil) |

| Affections cardiaques | |
| --- | --- |
| Fréquents | Bradycardie |
| Rares | Asystolie/arrêt cardiaque, habituellement précédé de bradycardie a été rapporté chez des patients ayant  reçu du rémifentanil en association avec d'autres agents anesthésiques. |
| **Affections vasculaires** | |
| Très fréquents | Hypotension |
| Fréquents | Hypertension postopératoire |
| **Affections respiratoires, thoraciques et médiastinales** | |
| Fréquents | Dépression respiratoire aiguë, apnée |
| Peu fréquents | Hypoxie |
| **Affections gastro-intestinales** | |
| Très fréquents | Nausées, vomissements |
| Peu fréquents | Constipation |
| **Affections de la peau et du tissu sous-cutané** | |
| Fréquents | Prurit |
| **Troubles généraux et anomalies au site d'administration** | |
| Fréquents | Frissons postopératoires |
| Peu fréquents | Douleurs postopératoires |

### Procédures mises en place en vue de l’enregistrement et de la notification des événements indésirables

###### Responsabilités de l’investigateur

Recueil des événements indésirables

Tous les événements indésirables seront notifiés sur les formulaires de recueil des événements indésirables du cahier d’observation. Chaque événement indésirable observé sera consigné individuellement. L’intensité des événements indésirables sera déterminée de la façon suivante

- - légère (grade 1) : pas d’interférence sur l’activité au quotidien du patient ;
  - modérée (grade 2) : interférence modérée sur l’activité quotidienne du patient mais encore acceptable ;
  - sévère (grade 3) : interférence importante sur l’activité quotidienne du patient et inacceptable ;
  - menace du pronostic vital (grade 4) ;
  - décès (grade 5).

Tous les événements indésirables doivent être gradés et évalués. Notification des événements indésirables graves

L’investigateur doit notifier au promoteur, sans délai à compter du jour où il en a connaissance, tous les événements indésirables graves survenant pendant la période de l’essai, à l'exception de ceux qui sont recensés dans le protocole comme ne nécessitant pas de notification.

Tous les événements indésirables graves devront faire l’objet d’un rapport sur un formulaire “ Evénement Indésirable Grave ” figurant dans le cahier d’observation.

Ce formulaire devra être transmis au promoteur (Cellule Vigilance de la Fédération de Recherche Clinique) par fax au 03 20 44 57 11.

Pour chaque événement indésirable, l’investigateur doit documenter au mieux l’événement et reporte :

- la description claire et détaillée de l’événement, sous forme de diagnostic médical si cela est possible
- la gravité, les dates de début et de fin de l’événement, son évolution
- le lien de causalité entre cet événement indésirable grave et le dispositif médical ou son geste de mise en œuvre.

Le suivi des événements indésirables sera assuré par l'investigateur.

L’investigateur doit joindre pour chaque EIG, de façon anonymisée, et à chaque fois que cela est possible :

- une copie du compte-rendu d’hospitalisation ou de prolongation d’hospitalisation
- une copie de tous les résultats d’examens complémentaires réalisés pertinents
- tout autre document qu’il jugera utile et pertinent

Période de notification des événements indésirables graves

Tout EIG doit être notifié, s’il survient pour un participant à la recherche :

- - à partir de la date de signature du consentement,
  - pendant toute la durée de suivi du participant prévue par l’essai,
  - et jusqu’à la fin de participation à l’essai du sujet,
  - sans limitation de durée lorsqu’il est susceptible d’être dû à la recherche / au(x) médicament(s) expérimental (aux) / au(x) dispositif(s) testé (s) (par exemple des effets graves pouvant apparaître à grande distance de l’exposition au médicament, tels des cancers ou des anomalies congénitales).

Signalement des grossesses

Une grossesse ne constitue pas un événement indésirable grave, néanmoins la survenue de celle-ci en cours d’essai doit être notifiée sans délai, sur le formulaire standard de signalement de grossesse, au promoteur qui s’assurera de son bon déroulement si jugé nécessaire.

L’investigateur devra suivre la patiente jusqu’au terme de la grossesse ou de son interruption et en notifier l’issue au promoteur grâce au formulaire standard de suivi de l’issue de la grossesse.

Si l’issue de la grossesse entre dans le cadre de la définition des événements indésirables graves (avortement spontané avec hospitalisation, mort fœtal, anomalie congénitale, ...) l’investigateur doit alors suivre la procédure de notification des EIG.

###### Responsabilités du promoteur

Déclaration des effets indésirables graves et inattendus

Pour chaque événement ou effet indésirable grave, le promoteur évalue la gravité et lien de causalité entre l’événement ou l’effet indésirable et le(s) produit(s) à l’étude, ou le protocole, ainsi que le caractère inattendu.

Le promoteur déclare à l'ANSM et au CPP toute **suspicion d'effet indésirable grave et inattendu** :

- dans le cas **d'effet indésirable grave inattendu ayant entraîné la mort ou mis la vie en danger**, sans délai à compter du jour où le promoteur en a eu connaissance ;
- dans le cas des **autres effets indésirables graves inattendus**, au plus tard dans un délai de quinze jours à compter du jour où le promoteur en a eu connaissance.

Le promoteur déclare sous forme d'un rapport de suivi à l'ANSM et au CPP les informations complémentaires pertinentes concernant :

- les suspicions d'effet indésirable grave inattendu ayant entraîné la mort ou mis la vie en danger, dans un délai de huit jours à compter du jour où le promoteur en a eu connaissance.
- les autres cas de suspicion d'effet indésirable grave inattendu, dans un nouveau délai de huit jours à compter du délai de quinze jours relatif à la déclaration initiale.

Déclaration des faits nouveaux de sécurité

En cas de survenue d’un fait nouveau de sécurité durant cette étude, le promoteur adressera immédiatement, par email, dès qu’il en a connaissance, une déclaration de ce fait nouveau et des éventuelles mesures prises, au CPP et à l’ANSM.

Rapport annuel de sécurité

Une fois par an pendant toute la durée de l’essai, ou sur demande, le promoteur transmet à l’ANSM et au CPP un **rapport de sécurité.** Ce rapport de sécurité comprendra notamment une analyse globale du profil de sécurité du protocole de l’étude prenant en compte toutes les nouvelles données pertinentes de sécurité. Les informations de sécurité apparaitront sous forme de tableaux de synthèse résumant les événements ou effets indésirables graves survenus dans la recherche biomédicale.

## Droit d’accès aux données et documents sources

L’investigateur s’engage à accepter les contrôles du promoteur (moniteur et/ou auditeur), ou de l'inspecteur de l’autorité administrative compétente. Il garantit l’accès aux données sources (dossiers médicaux, fichiers informatiques, documents de l’étude…).

## Contrôle et assurance de la qualité

La démarche Assurance Qualité qui sera mise en œuvre permet de prendre en charge les sujets se prêtant aux recherches dans les meilleures conditions de sécurité et de respect des règles médico- réglementaires.

- Déroulement de l'essai

Les observations médicales seront conservées dans le dossier du patient, les données concernant l'étude seront reportées sur les cahiers d'observation prévus pour l'étude, selon les bonnes pratiques cliniques, reprenant les différentes étapes de la prise en charge du patient dans le protocole. Tout écart au protocole sera notifié ainsi que sa raison. Le recueil des données devra être exhaustif et sera régulièrement vérifié par un Assistant de Recherche Clinique selon les procédures du protocole (si applicable).

- Monitoring de l’étude

Le monitoring de l’essai sera effectué selon le planning de monitoring validé avant le début de la recherche ou sur déclenchement spécifique par un ARC du promoteur. Il sera fonction des inclusions réalisées dans l’étude.

Une réunion de mise en place avec l’investigateur principal aura lieu avant le début de l’essai (rappel des BPC, organisation de la recherche, monitoring prévu).

L’investigateur informe le promoteur en temps réel des inclusions réalisées.

Lors des visites de monitoring sur site, les ARC devront pouvoir consulter :

- les cahiers de recueil de données des patients inclus
- les dossiers médicaux et infirmiers des patients
- le classeur investigateur

Le monitoring vérifiera au minimum les 6 points suivants :

- l’existence des patients, l’information et la présence des consentements éclairés signés
- le respect des critères d’inclusion
- le critère principal de jugement
- la surveillance et la déclaration des EIG
- la survenue de faits nouveaux nécessitant le dépôt d’un amendement
- La gestion et le contrôle des dispositifs médicaux
- Clôture de l'étude

A la fin de l'essai, des procédures de clôture seront appliquées, avec classement de tous les documents et des données sources. Une fois l'analyse finale effectuée et validée, l'ensemble du dossier et des données sont scellés et archivés selon des procédures spécifiques dans des locaux sécurisés.

## Considérations éthiques et légales

L'essai sera mené conformément au protocole approuvé, conformément au code de la Santé Publique, au BPC de l'UE et aux exigences réglementaires applicables. L’essai sera enregistré sur la base de données publique ClinicalTrials.gov.

### Comité de protection des personnes et Autorité compétente

###### Avis favorable du CPP

Le promoteur soumet une demande d’avis auprès du CPP avant le début de la recherche, conformément à l’article L1121-4 du Code de la Santé Publique. Le promoteur adresse une copie et un résumé de la recherche à l’ANSM.

###### Modifications au protocole

Le promoteur est seul autorisé à modifier le protocole, en concertation avec l’investigateur principal.

On entend par modifications substantielles, les modifications qui ont un impact significatif sur tout aspect de la recherche, notamment sur la protection des personnes, y compris à l’égard de leur sécurité, sur les conditions de validité de la recherche le cas échéant sur la qualité et la sécurité des produits expérimentés, sur l’interprétation des documents scientifiques qui viennent appuyer le déroulement de la recherche ou sur les modalités de conduite de celle-ci.

Une demande de modification substantielle est adressée par le promoteur au CPP. Dès réception de l’avis favorable, la version amendée du protocole est alors transmise par le promoteur, à l’ANSM pour information et à tous les investigateurs.

Une modification non substantielle du protocole est une modification mineure ou une clarification sans retentissement sur la conduite de l’essai. Ces modifications ne seront pas soumises aux autorités compétentes mais feront l’objet d’un accord entre le promoteur et l’investigateur et seront clairement documentées (dans le dossier de suivi de l’étude).

### Information et consentement

Conformément à la réglementation en vigueur, le participant à l’essai devra recevoir une information loyale, complète, à travers la Lettre d’Information, spécialement rédigée pour l’essai et validés par le Comité de Protection des Personnes, qui lui sera obligatoirement fournie et expliquée par l’Investigateur. Ce dernier devra notamment informer le participant des risques et contraintes éventuelles de la participation à l’essai.

Le participant pourra poser toute question lui semblant utile à sa réflexion, et disposera du temps nécessaire de réflexion afin de prendre sa décision en toute connaissance de cause.

Le participant à l’essai devra ensuite signer le formulaire de consentement, avec l’Investigateur lui ayant présenté l’essai, et ce document devra être daté du jour de la signature. Une copie sera remise au participant, une conservée par l’Investigateur, et une conservée dans le dossier médical du participant.

L’Investigateur devra s’assurer du respect des critères d’inclusion et d’exclusion avant l’inclusion du participant. Aucun acte spécifique à la recherche ne pourra avoir lieu avant l’information et l’obtention du consentement du participant à l’essai. A tout moment, le participant pourra retirer son consentement, et toute information sur la sécurité de leur participation devra leur être rapportée.

Inscription au fichier national des personnes qui se prêtent à une recherche biomédicale

Il n’y a pas d’inscription au fichier national des personnes qui se prêtent à une recherche biomédicale car l’étude a un rapport avec l’état pathologique et les personnes ne reçoivent pas d’indemnités.

## Traitement des données et conservation des documents et des données relatives à la recherche

Le traitement des données sera réalisé dans les conditions de confidentialité définies par la loi du 6 janvier 1978 modifiée relative à l’informatique, aux fichiers et aux libertés (CNIL). Le traitement des données sera effectué conformément aux exigences de la méthodologie de référence MR 06001 de la CNIL.

Afin de répondre au mieux aux objectifs de cette étude, l’ensemble des variables sera rédigé collégialement avec l’investigateur, le datamanager et les biostatisticiens.

Le CRF papier sera ensuite mis en forme afin d’organiser ces données et de permettre un recueil logique en fonction du parcours patient ou de la localisation des données à collecter.

La version finale du CRF sera validée par l’investigateur, le datamanager et les biostatisticiens.

Cette version finale permettra de développer un eCRF par l’Unité de Méthodologie, Biostatistiques et Datamanagement du Pr Duhamel au CHRU de Lille.

L’eCRF sera développé sur Ennov Clinical, agréé par la FDA et permettant une qualité de base de données optimale.

Cet eCRF permettra d'effectuer des tests à la volée en rapport avec le dictionnaire des données attendu ainsi que des tests de cohérence des données. Ces tests seront définis en amont du développement du masque de saisie par l'investigateur et le datamanager.

Un monitoring final des données, défini par les tests de cohérence mis en place, sera effectué afin de vérifier la cohérence et la qualité des données saisies.

Les données seront collectées directement sur l’eCRF. L’anonymisation des patients sera gérée en dehors de l’eCRF.

Le centre devra détenir la liste des correspondances identifiant / identité du patient de manière sécurisée et non accessible aux personnes n’ayant pas les droits de consultation. Ces identifiants seront reportés sur l’eCRF.

Les données seront hébergées au CHRU de Lille, via notre partenaire eSIS (Groupement d'Intérêt Public nommé GIP e-SiS 59/62)

Les données concernant cette étude seront archivées pendant une durée minimum de quinze ans à compter de la fin de la recherche ou de son arrêt anticipé sans préjudice des dispositions législatives et réglementaires en vigueur.

## Financement et assurance

### Financement

Les dispositifs médicaux sont fournis gratuitement par la société MetroDoloris®.

### Assurance

Le promoteur souscrit un contrat d’assurance garantissant sa responsabilité civile et celle de tout intervenant à l’étude, conformément à l’article L1121-10 du Code de la Santé Publique.

## Publication- Valorisation

Conformément à l’article R 5121-13 du Code de la Santé Publique, les essais ne peuvent faire l’objet d’aucun commentaire écrit ou oral sans l’accord conjoint de l’investigateur et du promoteur. Toute publication doit mentionner que le CHRU de Lille est promoteur (No d’identification “ 2010_51 ” à demander au Département de la Recherche en Santé). En tout état de cause, le CHRU de Lille, promoteur de l’étude, a la maîtrise de la première publication. L’investigateur adresse une copie de ses publications au promoteur.

Le promoteur est le propriétaire exclusif des résultats de l’étude. Ces résultats, ainsi que toutes les données relatives à la recherche, ne doivent en aucun cas être transmises à un tiers, sans contrepartie négociée préalablement par le Département de la Recherche en Santé. Toute sollicitation de ce type doit être transmise le plus tôt possible aux affaires juridiques du Département de la Recherche en Santé.

## Liste des annexes

**Annexe I** : Régulation de l’anesthésie basée sur l’ANI : description du dispositif ANI-LOOP

**Annexe II** : Etat de l'art sur les dispositifs d'administration en boucle des anesthésiques intraveineux

# Annexe I: Régulation de l’anesthésie basée sur l’ANI : description du dispositif ANI-LOOP

*ANI computation*

The ECG is digitized at a sampling rate of 250 Hz. ECG R waves are then detected in order to build the RR intervals series defined as the time evolution of the time intervals between two R waves. RR series is analyzed using an original non linear filtering algorithm in order to detect and replace each disturbed RR sample. Filtered RR series are then re-sampled at 8 Hz using a linear interpolation. RR series is then mean centered and normalized into a 64 seconds moving window. Since the method is based on the analysis of HF changes, the RR series is band pass filtered between [0.15-0.4 Hz]. The band pass filtering is realized using a numerical filter based on the 4 coefficients Daubechies wavelet.

Local maxima and minima are detected and the upper and lower envelopes are plotted by connecting the local minima together and the local maxima as well (red curves). The 64 sec moving window is then divided into four sub-windows of 16 sec. The areas between the lower and upper envelopes are then measured in the four sub-windows. We defined AUCmin as the smallest of these sub-areas.

ANI is then computed in order to obtain a value between 0 and 100:

ANI = 100 * [a*AUCmin+b] / 12.8 (1)

Where *a* = 5.1 and *b* = 1.2 have been empirically determined in a data set of more than 100 anesthetized patients in order to obtain a good correlation between the visual pattern of the parasympathetic influence on RR series and the quantitative measurement of ANI.

This parameter is available on the Physiodoloris® monitor commercialised by MetroDoloris® (Lille, France). The monitor user interface displays in real time the instantaneous and averaged values of the ANI index (respectively ANI_i_ in yellow and ANI_a_ in orange). The different index values are also available in real time through the classical RS-232 monitor serial port.

*B – Decision rules for analgesic drugs administration*

Since 2008, the Physiodoloris® monitor has been used in the orthopedic surgery unit of the Lille University hospital. We observed and recorded ANI evolution and reactions on more than 2000 patients during orthopedic surgical procedure under general anesthesia. Thanks to this experience, we determined several decision rules based on ANI_i_ and ANI_a_ analysis in order to help anesthesiologists understand ANI guided anesthesia. In order to regulate analgesic drug administration, we determined two types of action:

- ***Infusion flow Changes:*** Increase or decrease the syringe pump infusion rate.
- ***Bolus:*** Infusion rate fast increase during 10 s. Infusion rate returns to its previous value after these 10s.

Each action is followed by a *refractory period* which shuts down the regulation algorithm for several seconds.

Several regulation variables and constants are defined.

*Variables used for regulation:*

- ***ANI_i_ :*** instantaneous ANI.
- ***ANI_a_ :*** average ANI.
- ***S_i_:*** ANI_i_ slope (computed on 30 s).
- ***S_a_:*** ANI_a_ slope (computed on 30 s).
- ***InF:*** Syringe pump infusion Flow (in µg/Kg/min).

*Constants used for regulation (set by the user):*

- ***ANI_max_:*** representing the ANI max value.
- ***ANI_min_:*** representing the ANI min value.
- ***S_a_T:*** representing the S_a_ threshold
- ***S_i_T:*** representing the S_i_ threshold
- ***InI:*** Infusion Rate increment (in µg/Kg/min).
- ***InF_min_:*** Minimum infusion rate (in µg/Kg/min).
- ***Inf_max_:*** Maximum infusion rate (in µg/Kg/min).
- ***BolusI:*** Infusion Flow bolus increment (in µg/Kg/min).
- ***RefP****:* Refractory period (in s)

***ANI_mean_*** is computed as

ANI_mean_ = ANI_min_+(ANI_max_-ANI_min_)/2 (2)

The defined rules are established in order to treat both acute pain and global analgesia level changes.

*Acute pain treatment:*

**If**(ANI_i_< ANI_min_)**and(**S_i_<-S_i_T)**then** Bolus (3) Bolus corresponds to an Infusion rate increase of *Bolus* µg/Kg/min during 10 s.

Infusion rate returns to its previous baseline after these 10s.

Each Bolus is followed by a 3*RefP refractory period.

*Analgesia level changes:*

## ANI_a_<ANI_min_:

In this area, we consider that analgesia is insufficient. This event is treated by increasing the infusion pump rate.

**If(**InF<InFmax)**and**(S_a_<SaT) **then** InF = InF+InI (4)

## ANI_min_ <ANIa< ANI_max_ :

In this area, we consider that analgesia in adequate. However, in order to anticipate, we consider the ANI_a_ slope (S_a_) changes in order to regulate the infusion rate.

**If**(ANI_a_<ANI_mean_)**and**(S_a_<-S_a_T)**and**(InF+InI<InF_max_)

**then** InF = InF+ InI (5)

**If**(ANI_a_> ANI_mean_)**and** (S_a_>S_a_T)**and**(InF- InI>InF_min_)

## ANIa>75 :

**then** InF = InF- InI (6)

In certain condition, we consider that if ANI is too high, ANS doesn’t respond to noxious stimuli. This effect could be explained by an opioid overdose which would require an infusion flow decrease.

**If**(S_a_>0)**and**(InF- InI>InF_min_)**then** InF = InF- InI (7) Each infusion rate change is followed by a RefP refractory period.

In order to avoid arterial hypotension (SBP<80 mmHg), we added two rules on systolic blood pressure (SBP).

**If** SBP<90 **then** InF = InFmin (8)

**If** SBP<80 **then** InF = 0 (9)

In the case of SBP<80 mmHg, the regulation algorithm is shut down until SBP recovers a value over 85 mmHg. Rules (8) and (9) have the highest level of priority.

*C – Algorithm implementation*

From a technical point of view, the system consists in a software (“controller”) implemented on a classical personal computer allowing to adapt in real time the syringe pump infusion rate according to ANI_i_ and ANI_a_ evolutions. ANI_i_ and ANI_a_ values are obtained from the Physiodoloris monitor communication interface and SBP is obtained from the Anesthesia monitor (Datex Ohmeda AS-5, GE Healthcare or

Intelliview® Philips^TM^). The syringe pump (Alaris GH, Cardinal health) and the two monitors are linked to the computer through a Keyspan® 4-port serial to USB adapter.

The software also allows users to enter patient characteristics (age, size, weight, ASA) and constants values used for regulation. Thanks to the interactive aspect of the controller, all the predefined constants can be adapted and manually changed during the regulation. Finally, a specific software interface (allows users to follow the variable's evolution, the syringe pump status and the regulation results. Direct syringe pump commands are also available in order to allow the anesthesiologists to manually adapt the syringe pump infusion flow or to shut down the automatic regulation.

Constants used for regulation are defined as follow:

- ***ANI_max_=75***
- ***ANI_min_=50***
- ***S_a_T=7***
- ***S_i_T=25***
- ***InI=0.01 µg/kg/min***
- ***InF_min_=0.05 µg/kg/min***
- ***Inf_max_=0.6 µg/kg/min***
- ***BolusI= 0.04 µg/kg/min***
- ***RefP=10 s***

Blood pressure is measured every 2.5 minutes.

## ANNEXE II : Etat de l'art sur les dispositifs d'administration en boucle des anesthésiques intraveineux

Le moniteur PhysioDoloris (MDoloris Medical Systems, Loos, France), marqué CE, commercialisé depuis 2010, est une mesure reconnue de l'état du système nerveux autonome qui permet l'évaluation du tonus parasympathique (paraS) en temps réel à partir du recueil non invasif de l'électrocardiogramme. Il affiche en continu l'Analgesia Nociception Index (ANI), indice correspondant à la quantité relative de tonus paraS, qui varie entre 0 et 100. Plusieurs études ont démontré que l'ANI permet une mesure de l'état de la balance antinociception/nociception au cours de l'anesthésie générale ^8,9,11-14^. Chez le patient conscient en salle de surveillance post-interventionnelle, après une anesthésie générale, l'ANI permet de mesurer le niveau de douleur ^15^, sauf après une anesthésie prolongée au sevoflurane ^16^. En dehors de la période péri opératoire, chez le patient conscient, l'ANI présente un lien significatif avec la douleur ressentie, chez l'enfant ^17^ comme chez l'adulte ^18^.

## Il est important de noter que l'étude proposée ne porte pas sur le moniteur PhysioDoloris. L'étude porte sur un dispositif médical innovant (cf. annexe II) qui permet l'administration automatisée de rémifentanil à partir d'éléments issus du monitorage hémodynamique habituel (fréquence cardiaque et pression artérielle) associés à la mesure fournie en continu par le moniteur PhysioDoloris (ANI).

L'administration automatisée de propofol pour l'induction et l'entretien de l'anesthésie générale (AG) est une technique utilisée par de nombreuses équipes aujourd'hui. Le signal cible à maintenir dans un intervalle prédéfini est dérivé de l'électroencéphalogramme (EEG) dans la grande majorité des cas : il s'agit de l'indice BiSpectral® (Aspect Medical Systems, MA) et de l'entropie de l'EEG (GE HealthCare). Deux dispositifs médicaux sont actuellement engagés dans la phase de marquage CE après avoir fait la preuve de concept pré-industrielle: EasyTIVA® (MedSteer, Suresnes, France) et McSleepy® (université McGill, Québec, Canada)^19,20^. Un troisième dispositif médical d'administration de l'anesthésie générale totale intraveineuse (TIVA) en boucle fermée est marqué CE et commercialisé depuis 2016, le Concert-CL® (Veryark® Technology Co., Ltd., Guangxi, Chine)^21,22^.

La composante analgésique de l'AG a fait l'objet de progrès importants depuis une dizaine d'années avec la commercialisation de plusieurs moniteurs du système nerveux autonome (SNA) qui ont été utilisés pour mesurer la balance anti-nociception/nociception au cours de l'AG^23-31^. L'absence de signal physiologique spécifique de la nociception constitue la difficulté technique principale de la mise au points d'une boucle de rétrocontrôle pour l'administration de l'analgésie morphinique. Certains auteurs ont démontré que la pression artérielle moyenne pouvait être utilisée comme cible d'une administration d'alfentanil en boucle fermée^32^, tandis que d'autres ont mis au point un score spécifique, l'analgoscore, qui associe les variations de fréquence cardiaque et de pression artérielle (système McSleepy®)^19,20^. D'autres auteurs enfin utilisent l'EEG et l'éveil cortical induit par les phénomènes nociceptifs pour distinguer les besoins du patient en hypnotiques et en morphiniques (système EasyTIVA®): ce dispositif a déjà fait l'objet d'évaluations nombreuses dans des contextes cliniques variés^33,34^, et à démontré un réel avantage pour l'évaluation des effets d'épargne morphinique engendrés par l'utilisation de dexmedetomidine^35^ ou des besoins morphiniques de populations particulières comme celle des patients souffrant d'obésité morbide^36^. Deux méta-analyses récentes soulignent la sécurité d'utilisation des dispositifs d'administration en boucle fermée, et dans certains cas leur supériorité par rapport aux systèmes d'administration manuels^37,38^.

**Bibliographie**

- 1. Aubrun F, Nouette Gaulain K, Fletcher D, Belbachir A, Beloeil H, Carles M, Cuvillon P, Dadure C, Lebuffe G, Marret E, Martinez V, Olivier M, Sabourdin N, Zetlaoui P: Réactualisation de la recommandation sur la douleur postopératoire. Revision of expert panel guidelines on postoperative pain management. Anesth Réanim 2016; 2: 421-30
  2. Pellat JM, Hodaj H, Alibeu JP, Payen JF, Jacquot C: Hyperalgésie postopératoire Description clinique, mécanismes et prévention. Douleurs 2006; 7: 11-16
  3. Richebe P, Pouquet O, Jelacic S, Mehta S, Calderon J, Picard W, Rivat C, Alex Cahana A, Janvier G: Target-Controlled Dosing of Remifentanil During Cardiac Surgery Reduces Postoperative Hyperalgesia. J Cardiothorac Vasc Anesth 2011; 25: 917-25
  4. Task_Force: Heart rate variability. Standards of measurement, physiological interpretation and clinical use. Task Force of the European Society of Cardiology and the North American Society of Pacing and Electrophysiology. Circulation 1996; 93: 1043-65
  5. Jeanne M, Logier R, De Jonckheere J, Tavernier B: Heart rate variability during total intravenous anesthesia: effects of nociception and analgesia. Auton Neurosci 2009; 147: 91-6
  6. Jeanne M, Logier R, De Jonckheere J, Tavernier B: Validation of a graphic measurement of heart rate variability to assess analgesia/nociception balance during general anesthesia. Conf Proc IEEE Eng Med Biol Soc 2009; 1: 1840-3
  7. Jeanne M, Clement C, De Jonckheere J, Logier R, Tavernier B: Variations of the analgesia nociception index during general anaesthesia for laparoscopic abdominal surgery. J Clin Monit Comput 2012; 26: 289-94
  8. Gruenewald M, Ilies C, Herz J, Schoenherr T, Fudickar A, Höcker J, Bein B: Influence of nociceptive stimulation on analgesia nociception index (ANI) during propofol-remifentanil anaesthesia. Br J Anaesth 2013; [Epub ahead of print]
  9. Sabourdin N, Arnaout M, Louvet N, Guye ML, Piana F, Constant I: Pain monitoring in anesthetized children: first assessment of skin conductance and analgesia-nociception index at different infusion rates of remifentanil. Paediatr Anaesth 2013; 23: 149-55
  10. Jeanne M, de jonckheere J, Butruille L, Logier R, Tavernier B: L'estimation de la balance analgésie/nociception avec l'indice ANI. OXYMAG 2015; 140
  11. Migeon A, Desgranges FP, Chassard D, Blaise BJ, De Queiroz M, Stewart A, Cejka JC, Combet S, Rhondali O: Pupillary reflex dilatation and analgesia nociception index monitoring to assess the effectiveness of regional anesthesia in children anesthetised with sevoflurane. Paediatr Anaesth 2013; 23: 1160-5
  12. Boselli E, Logier R, Bouvet L, Allaouchiche B: Prediction of hemodynamic reactivity using dynamic variations of Analgesia / Nociception Index (dANI). J Clin Monit Comput 2015; [Epub ahead of print]
  13. Boselli E, Bouvet L, Bégou G, Torkmani S, Allaouchiche B: Prediction of haemodynamic reactivity during total intravenous anaesthesia for suspension laryngoscopy using Analgesia/Nociception Index (ANI): a prospective observational study. Minerva Anestesiol 2015; 81: 288-97
  14. Boselli E, Logier R, Bouvet L, Allaouchiche B: Prediction of hemodynamic reactivity using dynamic variations of Analgesia/Nociception Index (ANI). J Clin Monit Comput 2016; 30: 977-984
  15. Boselli E, Daniela-Ionescu M, Bégou G, Bouvet L, Dabouz R, Magnin C, Allaouchiche B: Prospective observational study of the non-invasive assessment of immediate postoperative pain using the analgesia/nociception index (ANI). Br J Anaesth 2013; 111: 453-9
  16. Ledowski T, Tiong WS, Lee C, Wong B, Fiori T, Parker N: Analgesia nociception index: evaluation as a new parameter for acute postoperative pain. Br J Anaesth 2013; 111: 627-9
  17. Avez-Couturier J, De Jonckheere J, Jeanne M, Vallée L, Cuisset JM, Logier R: Assessment of Procedural Pain in Children Using Analgesia Nociception Index: A Pilot Study. clin J Pain 2016
  18. Le Guen M, Jeanne M, Sievert K, Al Moubarik M, Chazot T, Laloë PA, Dreyfus JF, Fischler M: The Analgesia Nociception Index: a pilot study to evaluation of a new pain parameter during labor. Int J Obstet Anesth 2012; 21: 146-51
  19. Charabati S, Bracco D, Mathieu PA, Hemmerling TM: Comparison of four different display designs of a novel anaesthetic monitoring system, the 'integrated monitor of anaesthesia (IMA)'. Br J Anaesth 2009; 103: 670-7
  20. Hemmerling TM, Arbeid E, Wehbe M, Cyr S, Taddei R, Zaouter C: Evaluation of a novel closed-loop total intravenous anaesthesia drug delivery system: a randomized controlled trial. Br J Anaesth 2013; 110: 1031-9
  21. Liu Y, Li M, Yang D, Zhang X, Wu A, Yao S, Xue Z, Yue Y: Closed-loop control better than open-loop control of profofol TCI guided by BIS: a randomized, controlled, multicenter clinical trial to evaluate the CONCERT-CL closed-loop system. PLoS ONE 2015; 10
  22. Liu Y, Li M, Yang D, Zhang X, Wu A, Yao S, Xue Z, Yue Y: Closed-loop control better than open-loop control of profofol TCI guided by BIS: a randomized, controlled, multicenter clinical trial to evaluate the CONCERT-CL closed-loop system. PLoS ONE 2015; 10
  23. Struys MM, Vanpeteghem C, Huiku M, Uutela K, Blyaert NB, Mortier EP: Changes in a surgical stress index in response to standardized pain stimuli during propofol-remifentanil infusion. Br J Anaesth 2007; 99: 359-67
  24. Wennervirta J, Hynynen M, Koivusalo AM, Uutela K, Huiku M, Vakkuri A: Surgical stress index as a measure of nociception/antinociception balance during general anesthesia. Acta Anaesthesiol Scand 2008; 52: 1038-45
  25. Bonhomme V, Uutela K, Hans G, Maquoi I, Born JD, Brichant JF, Lamy M, Hans P: Comparison of the Surgical Pleth IndexTM with haemodynamic variables to assess nociception–anti-nociception balance during general anaesthesia. Br J Anaesth 2011; 106: 101- 11
  26. Hans P, Verscheure S, Uutela K, Hans G, Bonhomme V: Effect of a fluid challenge on the Surgical Pleth Index during stable propofol-remifentanil anaesthesia. Acta Anaesthesiol Scand 2012; 56: 787-96
  27. Bergmann I, Göhner A, Crozier TA, Hesjedal B, Wiese CH, Popov AF, Bauer M, Hinz JM: Surgical pleth index-guided remifentanil administration reduces remifentanil and propofol consumption and shortens recovery times in outpatient anaesthesia. Br J Anaesth 2013; 110: 622-8
  28. Constant I, Nghe MC, Boudet L, Berniere J, Schrayer S, Seeman R, Murat I: Reflex pupillary dilatation in response to skin incision and alfentanil in children anaesthetized with sevoflurane : a more sensitive measure to noxious stimulation than the commonly used variables. Br J Anaesth 2006; 96: 614-9
  29. Isnardon S, Vinclair M, Genty C, Hebrard A, Albaladejo P, Payen JF: Pupillometry to detect pain response during general anaesthesia following unilateral popliteal sciatic nerve block: a prospective, observational study. Eur J Anaesthesiol 2013; 30: 429-34
  30. Larson MD, Behrends M: Portable infrared pupillometry: a review. Anesth Analg 2015; 120: 1242-53
  31. Constant I, Sabourdin N: Monitoring depth of anesthesia: from consciousness to nociception. A window on subcortical brain activity. Paediatr Anaesth 2015; 25: 73-82
  32. Luginbühl M, Bieniok C, Leibundgut D, Wymann R, Gentilini A, Schnider TW: Closed-loop control of mean arterial blood pressure during surgery with alfentanil: clinical evaluation of a novel model-based predictive controller. Anesthesiology 2006; 105: 462-70
  33. Liu N, Chazot T, Hamada S, Landais A, Boichut N, Dussaussoy C, Trillat B, Beydon L, Samain E, Sessler DI, Fischler M: Closed-loop coadministration of propofol and remifentanil guided by bispectral index: a randomized multicenter study. Anesth Analg 2011; 112: 546-57
  34. Orliaguet GA, Benabbes Lambert F, Chazot T, Glasman P, Fischler M, Liu N: Feasibility of closed-loop titration of propofol and remifentanil guided by the bispectral monitor in pediatric and adolescent patients: a prospective randomized study. Anesthesiology 2015; 122: 759-67
  35. Le Guen M, Liu N, Tounou F, Augé M, Tuil O, Chazot T, Dardelle D, Laloë PA, Bonnet F, Sessler DI, Fischler M: Dexmedetomidine reduces propofol and remifentanil requirements during bispectral index-guided closed-loop anesthesia: a double-blind, placebo- controlled trial. Anesth Analg 2014; 118: 946-55
  36. Liu N, Lory C, Assenzo V, Cocard V, Chazot T, Le Guen M, Sessler DI, Journois D, Fischler M: Feasibility of closed-loop co-administration of propofol and remifentanil guided by the bispectral index in obese patients: a prospective cohort comparison. Br J Anaesth 2015; 114: 605-14
  37. Pasin L, Nardelli P, Pintaudi M, Greco M, Zambon M, Cabrini L, Zangrillo A: Closed- Loop Delivery Systems Versus Manually Controlled Administration of Total IV Anesthesia: A Meta-Analysis of Randomized Clinical Trials. . Anesth Analg 2016
  38. Brogi E, Cyr S, Kazan R, Giunta F, Hemmerling TM: Clinical Performance and Safety of Closed-Loop Systems: A Systematic Review and Meta-Analysis of Randomized Controlled Trials. Anesth Analg 2016
