# Supplementary material for: Clinical efficacy and safety of automatic remifentanil administration based on Analgesia Nociception Index monitoring during burn surgery under propofol anesthesia: A randomized controlled clinical trial
Source: PLoS One. 2025 May 5;20(5):e0322384. doi: 10.1371/journal.pone.0322384 (PMC12052174; doi:10.1371/journal.pone.0322384)
Supplement: S5 File — (DOCX) [file pone.0322384.s005.docx]

"**Automatic ANI-guided remifentanil administration during propofol general anesthesia".**

### ANI-REMI-loop

Code number assigned by the promoter: **2016_48**

IDRCB N°: **2017-A00858-45**

| Research Involving the Human Person (RIPH) | Category 1 research: Interventional research that involves an intervention on a person that is not justified by the person's health.  usual care |
| --- | --- |

Principal investigator:

Dr JEANNE Mathieu

Clinique d'Anesthésie Réanimation Roger Salengro Boulevard Emile Laine

59037 LILLE cedex

Tel 03 20 44 40 74

Mail: [mathieu.jeanne@chru-lille.fr](mailto:mathieu.jeanne@chru-lille.fr)

Investigators :

Dr LAFANECHERE Aurélie

Clinique d'Anesthésie Réanimation Roger Salengro Boulevard Emile Laine

59037 LILLE cedex

tel 03 20 44 40 74

mail: [aurelie.lafanechere@chru-lille.fr](mailto:aurelie.lafanechere@chru-lille.fr)

Dr DEVAUCHELLE Pauline

Clinique d'Anesthésie Réanimation Roger Salengro Boulevard Emile Laine

59037 LILLE cedex

tel 03 20 44 40 74

mail: [Pauline.CLEMENTGRANDCOURT@CHRU-LILLE.FR](mailto:Pauline.CLEMENTGRANDCOURT@CHRU-LILLE.FR)

Scientific collaborator : DE JONCKHEERE Julien, Research Officer

INSERM CIC-IT 1403, MRRC

6 rue du professeur Laguesse 59037 LILLE Cedex

Tel 03 20 44 67 54

Mail: [julien.dejonckheere@chru-lille.fr](mailto:julien.dejonckheere@chru-lille.fr)

FLOCTEIL Mathilde, Clinical Study Technician INSERM CIC-IT 1403, MRRC

6 rue du professeur Laguesse 59037 LILLE Cedex

Tel 0320446754

Mail: [mathilde.flocteil@chru-lille.fr](mailto:mathilde.flocteil@chru-lille.fr)

Study sponsor : **Lille Regional & University Hospital Centre**

Department of Health Research (DRS) 6 rue Paul Laguesse

59037 LILLE Cedex

tel: 03 20 44 59 69

Current version no./Date: Version 2.0 of 19/01/2018

###### PROTOCOL SIGNATURE PAGE

**Developer code:** 2016_48

**Title long/short:** ANI-guided automatic piloting of remifentanil administration during propofol general anesthesia / ANI-REMI-loop.

**Version no. and date**: n°2.0 of 19/01/2018

The principal investigator and the sponsor undertake to carry out this study in accordance with the protocol, the rules and recommendations of international good clinical practice and the legal and regulatory provisions applicable to research.

| PROMOTER LEGAL REPRESENTATIVE | Lille University Hospital  Mr Frédéric BOIRON | DATE  _ _ / _ _ / _ _ _ _ | SIGNATURE |
| --- | --- | --- | --- |

###### SIGNATURE OF PRINCIPAL INVESTIGATOR

I have read all the pages of this protocol, for which the CHRU de Lille is the promoter, and I confirm that it contains all the information required to conduct the trial.

I undertake to carry out the trial in compliance with the protocol and the terms and conditions defined therein, as well as any amendments thereto that may be sent to me by the sponsor. I undertake to conduct this protocol in accordance with Good Clinical Practice, the Public Health Law of August 9, 2004 and the implementing decree of November 16, 2016, and in particular by providing information and obtaining written consent from patients prior to any protocol selection procedure.

I undertake to ensure that investigators and other qualified members of my team have access to copies of this protocol and documents relating to the conduct of the trial to enable them to work in compliance with the provisions set out in these documents.

I have been informed that my personal data will be processed automatically for the purpose of setting up and carrying out the research. This information may be transferred outside the European Union. In accordance with the amended French Data Protection Act of January 6, 1978, I have the right to access and rectify my personal data.

| PRINCIPAL INVESTIGATOR ESTABLISHMENT | Dr Mathieu JEANNE  Clinique d'Anesthésie Réanimation Roger Salengro Boulevard Emile Laine 59037 LILLE cedex | DATE  _ _ / _ _ / _ _ _ _ | SIGNATURE |
| --- | --- | --- | --- |

### CONTENTS

[List of abbreviations 5](#_bookmark0)

1. [SYNOPSIS 6](#_bookmark1)
2. [Rational: Scientific justification and general description of the research 9](#_bookmark2)
3. [Research objective 10](#_bookmark3)
   1. [Main objective 10](#_bookmark4)
   2. [Secondary objectives and hypotheses 10](#_bookmark5)
4. [Research design 10](#_bookmark6)
   1. [Experimental design 10](#_bookmark7)
   2. [Definition of the study population 11](#_bookmark8)
      1. [Inclusion criteria 11](#_bookmark9)
      2. [Non-inclusion criteria 11](#_bookmark10)
      3. [Exclusion criteria 12](#_bookmark11)
   3. [Evaluation criteria 12](#_bookmark12)
      1. [Definitions 12](#_bookmark13)
      2. [main criterion, making it possible to meet the main objective 12](#_bookmark14)
      3. [secondary criteria to meet secondary objectives: 12](#_bookmark15)
   4. [Calculating the number of subjects 13](#_bookmark16)
   5. [Analysis method and strategy 13](#_bookmark17)
5. [Study logistics 13](#_bookmark18)
   1. [Participating teams and team experience in the field 13](#_bookmark19)
   2. [Practical aspects of the research 14](#_bookmark20)
      1. [Informed consent 14](#_bookmark21)
      2. [Devices used for anesthesia 14](#_bookmark22)
      3. [Anesthesia procedure 14](#_bookmark23)
   3. [Duration 16](#_bookmark24)
   4. [Criteria for discontinuing study participation 16](#_bookmark25)
   5. [Simultaneous participation ban - Exclusion period 16](#_bookmark26)
   6. [Study benefits, risks and constraints 16](#_bookmark27)
      1. [PROFIT 16](#_bookmark28)
      2. [COLLECTIVE PROFIT 16](#_bookmark29)
      3. [RISKS 16](#_bookmark30)
      4. [Supervisory Board 16](#_bookmark31)
6. [Medical devices 17](#_bookmark32)
7. [Safety assessment 17](#_bookmark33)
   1. [Definition 17](#_bookmark34)
      1. [Undesirable event 17](#_bookmark35)
      2. [Unwanted effect 17](#_bookmark36)
      3. [Serious adverse event or reaction 17](#_bookmark37)
      4. [Unexpected adverse reaction 17](#_bookmark38)
      5. [New fact 17](#_bookmark39)
   2. [Description of safety assessment parameters and risks associated with the protocol 17](#_bookmark40)
   3. [Adverse events related to Propofol and Remifentanil 18](#_bookmark41)
      1. [Propofol-related adverse events 18](#_bookmark42)
      2. [Remifentanil-related adverse events 19](#_bookmark43)
   4. [Procedures for recording and reporting adverse events20](#_bookmark44)
      1. [Investigator's responsibilities 20](#_bookmark45)
      2. [Developer's responsibilities 21](#_bookmark46)

1. [Right of access to source data and documents 22](#_bookmark47)
2. [Quality control and assurance 22](#_bookmark48)
3. [Ethical and legal considerations 23](#_bookmark49)
   1. [Personal Data Protection Committee and Competent Authority 23](#_bookmark50)
      1. [Favourable opinion of the CPP 23](#_bookmark51)
      2. [Changes to the protocol 23](#_bookmark52)
   2. [Information and consent 23](#_bookmark53)
4. [Data processing and storage of documents and research data 24](#_bookmark54)
5. [Financing and insurance 25](#_bookmark55)
   1. [Financing 25](#_bookmark56)
   2. [Insurance 25](#_bookmark57)
6. [Publication- Valuation 25](#_bookmark58)
7. [List of appendices 26](#_bookmark59)

#### List of abbreviations

| **ABBREVIATION** | **DEFINITION** |
| --- | --- |
| ANI | Analgesia Nociception Index |
| ANSM | Agence Nationale de Sécurité du Médicament et des Produits de Santé (French Agency for the Safety of Medicines and Health Products) |
| ARC | Clinical Research Associate |
| ASA | American Society of Anestheology |
| CHRU | Regional University Hospital of Lille |
| CNIL | Commission Nationale de l'Informatique et des Libertés (French Data Protection Authority) |
| PPC | Comité de Protection des Personnes |
| CRF/eCRF | Case Report Form/ electronic Case Report Form |
| EI | Undesirable Event |
| EIG | Serious Undesirable Event |
| EVA | Visual Analog Scale |
| FC | Heart Rate |
| FDA | Food and Drug Administration |
| NOT | Systolic blood pressure |
| PK / PD | Pharmacokinetics / pharmacodynamic |
| Remi | Remifentanil |
| SNA | Autonomic Nervous System |
| VFC | Heart Rate Variability |

1. **SYNOPSIS**

| PROMOTEUR | **Lille University Hospital**  Health Research Department  6 rue du Pr Laguesse - 59037 Lille Cedex Tel: 03 20 44 41 45 - Fax: 03 20 44 57 11 |
| --- | --- |
| TITLE | Automatic ANI-guided remifentanil administration during propofol general anesthesia |
| PRINCIPAL INVESTIGATOR | Dr Mathieu JEANNE |
| NUMBER OF CENTERS | 1 Center: Burns Treatment Center, Roger Salengro Hospital, Lille CHRU |
| TYPE OF STUDY | Controlled, Randomized, Single-blind, Class 1 single-center |
| OBJECTIVES | **Main objective**  To determine whether the ANI-loop medical device reduces the total amount of remifentanil (remi) administered compared with standard practice.  **Secondary objectives**  To determine whether automated administration of remifentanil by the ANI-loop device allows :   - improving hemodynamic stability during propofol general anesthesia. - increasing the time spent with an ANI between 50 and 70. - reducing immediate post-operative pain. |
| EXPERIMENTAL DESIGN | - category 1 randomized controlled trial - monocentric: Lille CHRU burn treatment center - randomization carried out by the Medical Promotion Department directly via the electronic CRF - in the *"ANI-loop"* arm, autonomous administration of remifentanil by the ANI-loop medical device (class IIb) under the supervision of an anesthetist - in the "standard practice" arm, administration of remifentanil adapted by the anaesthetist according to usual practice - ANI monitor visible on both arms |
| EVALUATION CRITERIA | **Main criterion:**  Total quantity of remi administered (value related to weight and duration of intervention).  **Secondary criteria to meet secondary objectives:**   - Proportion of time spent in hemodynamic reactivity, hypotension or bradycardia during the procedure. This proportion is equal to the cumulative duration of each episode of reactivity, hypotension or bradycardia, divided by the duration of the procedure. - Total dose of Ephedrine - ANIi and ANIm: proportion of time spent during |

|  | intervention in the following ranges: <50, [50-70], >70   - hypnotic component measurement index (BIS® or Entropie®): proportion of time spent during the procedure in the following intervals: <40, [40-60], >60 - ANI, heart rate, blood pressure, FetCO2, Pmax (ventilator) 5 min before start of procedure, 5 min after start of procedure and 20 min after start of procedure. - Pain assessed by visual analog scale (VAS) between H0 and H2, every 15 min.   Total quantity of morphine administered between end of surgery and H2 post-op.   - Total quantity of ketamine administered between end of surgery and H2 post-op.   -Frequency of postoperative nausea/vomiting assessed at H2.  Number of changes in remifentanil flow rate during the procedure   - Total quantity of propofol administered (absolute value, value related to weight, value related to weight and duration of procedure). - Number of propofol target changes during the procedure   In the *"ANI-loop"* arm only: number and total duration of disengagements of the ANI-loop device during the operation, i.e. the number of times and cumulative duration that the closed-loop system had to be interrupted and replaced by manual control as in the standard practical arm. |
| --- | --- |
| INCLUSION CRITERIA | - patients treated at the Lille CHRU Burn Treatment Centre - burn surgery: excision-grafting - age ≥ 18 years - informed consent - ASA I or II or III status - general anaesthesia with intubation and controlled assisted ventilation - body mass index between 17 and 40 kg.m-2 |
| NON-INCLUSION CRITERIA | - pregnant or breast-feeding women (simple questioning) - pace maker (regardless of operating mode) - non-sinus rhythm - heart transplantation - dysautonomia - diabetes mellitus with micro or macro angiopathic complications - foreseeable allergy or intolerance to a drug used in the study - baseline" hemodynamic measurements considered "reactivity", hypotension or bradycardia - FCbl>120 min^-1^ or PASbl>160 mmHg - uninsured - persons deprived of their liberty, in emergency situations, or persons incapable of consent and not benefiting from a legal protection regime (guardianship/curatorship) - unconscious or sedated patient before induction |
| NUMBER OF PARTICIPANTS | The aim of the study was to assess whether the ANI-loop medical device reduces the total amount of remifentanil administered compared with standard practice. On the basis of data in the literature [Leal PC et al.] and the usual practice of the |

|  | service, the total amount of remifentanil in the control group can be estimated at 0.30 ± 0.075 (mean ± standard deviation). To demonstrate a 20% reduction in the *ANI-loop* arm (i.e. a mean remifentanil quantity of 0.24 in the experimental group), with a first-species risk of 5% and a power of 80%, it is necessary to include 26 patients per group, for a total of 52 patients. |
| --- | --- |
| STATISTICAL ANALYSIS STRATEGY | Statistical analyses will be carried out using SAS software (version 9.4 or higher) and conducted at the Lille CHRU methodological support platform under the responsibility of Pr A. Duhamel. To meet the primary objective, the total amount of remifentanil administered (value related to weight and duration of intervention) will be compared between the two study arms using a Student's t-test; the effect size will be calculated (standardized mean difference) with a 95% confidence interval. If the distribution of the primary endpoint deviates from normality, the Mann-Whitney U test will be used. |
| INVESTIGATION PROCEDURE SPECIFIC TO THE STUDY AND DIFFERENCES FROM USUAL CARE | - standard practice" arm: management is identical to that of total intravenous general anaesthesia - *ANI-loop"* arm: management differs from standard practice only in the administration of remi, which is administered via the ANI-loop device. |
| ASSESSING THE BENEFITS AND RISKS OF RESEARCH | PROFIT  Use of the device should result in more stable hemodynamics and therefore better anesthesia quality, as well as potentially lower total remi administration.  RISK   - standard practice" arm: the risk is identical to that of any general anaesthetic - *ANI-loop"* arm: in the event of failure of the ANI-loop device, the anesthetist can disengage it at any time and continue anesthetic management according to "standard practice" without delay. The risk is therefore comparable to that of the "standard practice" arm. |
| EXCLUSION PERIOD | Simultaneous participation in another study is prohibited, as it would run the risk of interacting with autonomic nervous system responses. This prohibition is limited to the duration of the study, i.e. the perioperative phase. |
| REASONS FOR SETTING UP OR NOT SETTING UP A SUPERVISORY COMMITTEE | No, in view of the similar risk in the *"ANI-loop"* arm and in the "standard practice" arm, the creation of a monitoring committee does not seem justified. |
| DURATION OF STUDY | - Length of inclusion period: 12 months - Duration of study participation for one subject: from arrival in the OR to H2 post-op. - Duration of research: 15 months - Data analysis time: 3 months |

#### Rational: Scientific justification and general description of the research

Pain management in the hospital environment is an integral part of improving the quality of care. The difficulty for caregivers lies in assessing the level of pain in order to adapt prescriptions and limit the risk of adverse effects from these drugs^1^ . General anesthesia combines hypnotic and analgesic drugs to enable surgical procedures to be performed. The clinical and paraclinical data, such as pulse and blood pressure, used to monitor patients under general anesthesia do not enable us to accurately anticipate patients' analgesic requirements. What's more, the potent drugs used expose patients to the ever-present risk of overdosing, making it necessary to seek out the minimum doses required for the surgical procedure. On the other hand, inadequate analgesia could have cardiovascular repercussions (in particular, increased heart rate or blood pressure), which would be detrimental to the surgical procedure (increased bleeding) and could lead to heart attacks in coronary patients, as well as complications during the recovery phase (pain, anxiety). The quality of anesthesia depends on the optimal use of anesthetic products, which prevent the patient, even when unconscious, from feeling the pain associated with surgery. The search for the minimum effective dose of analgesics is also a major objective of modern anesthesia, in order to limit the risk of post-operative hyperalgesia^2,3^ . Optimizing the administration of analgesics can be expected to improve recovery conditions and reduce the risk of peri-operative complications, which could contribute to the evolution of clinical practices in favor of shorter hospital stays.

Studies have clearly demonstrated that cardiovascular regulatory processes are closely linked to pain perception. Heart rate variability (HRV) analysis is a well-known, non-invasive method for measuring the regulation of the cardiovascular system by the autonomic nervous system (ANS). This analysis is based on the principle that heart rate fluctuations reflect the activity of the sympathetic and parasympathetic systems, the main components of the ANS^4^ . Studies have shown that heart rate fluctuations above 0.15 Hz are exclusively due to the influence of the parasympathetic system. In adults, painful, fearful or anxious phenomena are accompanied by a decrease in high-frequency HRV (>0.15 Hz), indicating a reduction in parasympathetic tone during unpleasant stimuli or emotions. During surgery, HRV analysis correlates with the balance between nociception and analgesia. In children, a significant decrease in high-frequency spectral power has been observed during painful surgical procedures, suggesting a reduction in parasympathetic influence during the nociceptive stimulus. The Lille team, which includes clinicians and scientists associated with the Clinical Investigation Center - Technological Innovation (INSERM CIC-IT 1403) at the Lille CHRU, has been working on HRV analysis for several years. This work has led to the creation of an original HRV index linked to the analgesia-nociception balance: the ANI (Analgesia Nociception Index). This technology has been the subject of several patent applications. This technology is now available through the Physiodoloris® device (Mdoloris Medical Systems®, Loos, France). This CE-marked medical device is currently in routine clinical use in numerous hospitals .^5-9^

Recent studies have shown that using the Physiodoloris® device to adapt the analgesic component of general anaesthesia can limit the occurrence and duration of reactive episodes that are deleterious to the patient, while significantly reducing intraoperative morphine consumption. In most of these studies, the aim was to regulate remifentanil administration by manually changing the flow rate of the syringe pump according to the displayed ANI values. Thanks to our experience in the use of ANI during general anesthesia, we have established various decision rules based on ANI and blood pressure analysis, to guide the anesthetist in the management of intraoperative analgesia.

Our latest innovation is a medical device that automates the administration of remifentanil according to a pre-established algorithm. Two patents have been filed for this innovation. The ANI-loop medical device takes the form of a controller implemented on a microcomputer, connected to the multiparameter scope used for anesthesia monitoring, as well as to the Physioldoloris® monitor and an electric syringe pump (Appendix I).

We propose to conduct a randomized controlled trial to determine whether use of the ANI-loop medical device during propofol general anesthesia reduces the total dose of remifentanil administered.

#### Research objective

##### Main objective

To determine whether the ANI-loop medical device reduces the total amount of remifentanil (remi) administered compared with standard practice.

##### Secondary objectives and hypotheses

To determine whether the automated administration of remifentanil by the ANI-loop device allows:

- improving hemodynamic stability during propofol general anesthesia.
- increasing the time spent with an ANI between 50 and 70.
- reducing immediate post-operative pain.

In the *"ANI-loop*" arm only: determine whether ANI-loop enables fully automated administration (i.e. without the need to disengage the device).

#### Research design

##### Experimental design

- category 1 randomized controlled trial versus standard practice
- monocentric: Lille university hospital burn treatment center. Randomization carried out by the data management division directly via the electronic CRF
- in the *"ANI-loop"* arm, autonomous administration of remifentanil by the ANI- loop medical device under the supervision of an anesthetist
- in the "standard practice" arm, administration of remifentanil adapted by the anaesthetist according to usual practice
- ANI monitor visible on both arms

##### Definition of the study population

The ANI measurement provided by the PhyioDoloris monitor cannot be interpreted in several situations. All the criteria concerned are taken into account in the non-inclusion or exclusion criteria;

- arrhythmia
  - Exclusion criteria
- absence of breathing (e.g. apnea due to intubation)
  - Not applicable; the ANI-loop device is not used in automatic mode when the patient is not intubated and mechanically ventilated.
- respiratory rate below 9 cycles/min
  - Not applicable; the protocol provides for a respiratory rate of 12 minute cycles.
- current volume variable over the measurement time, i.e. 64 seconds
  - Not relevant; for this type of anaesthesia, tidal volume adjustments are usually made at most 1 to 2 times per hour. Significant changes in

tidal volume over a period of 64 seconds are not used in this clinical context.

- irregular breathing (when talking, laughing or coughing)
  - Not applicable; the ANI-loop device is not used in automatic mode when the patient is not intubated and mechanically ventilated.
- pace maker (certain types)
  - Non-inclusion criteria
- heart transplantation
  - Non-inclusion criteria
- Use of drugs affecting cardiac sinus activity (Atropine...)
  - Excusion criteria; atropine injections(parasympatholytic) andpropranolol (sympatholytic)
    1. Inclusion criteria
- patients treated at the Lille CHRU Burn Treatment Centre
- burn surgery: excision-grafting
- age ≥ 18 years
- informed consent
- ASA I or II or III status
- general anaesthesia with intubation and controlled assisted ventilation
- body mass index between 17 and 40 kg.m-2
  - 1. Non-inclusion criteria
- pregnant or breast-feeding women (simple questioning)
- pace maker (regardless of operating mode)
- heart transplantation
- non-sinus rhythm
- dysautonomia
- diabetes mellitus with micro or macro angiopathic complications
- foreseeable allergy or intolerance to a drug used in the study
- baseline" hemodynamic measurements considered as hypotension or bradycardia
- FCbl>120 min^-1^ or PASbl>160 mmHg
- uninsured
- persons deprived of their liberty, in emergency situations, or persons incapable of consent and not benefiting from a legal protection regime (guardianship/curatorship)
- unconscious or sedated patient before induction

NB: There is no pregnancy test to check this criterion; it will be a simple questioning.

- - 1. Exclusion criteria
- Arrhythmia
- significant extrasystole (more than two extrasystoles per minute without interruption during surgery)
- Intraoperative atropine injection
- Intraoperative injection of propranolol.

If a patient is excluded, his or her inclusion is deemed null and void; the investigating center will then proceed with an additional inclusion in replacement. No follow-up of the excluded patient is justified by participation in the study.

##### Evaluation criteria

- - 1. Definitions
- *baseline" values* for heart rate (HRbl) and systolic blood pressure (SBPbl) are the mean of 3 measurements taken before the start of anesthesia
- *hemodynamic reactivity*: HR > 120% of HRbl or SBP > 120% of SBPbl
- *hypotension*: PAS <75 mmHg
- *bradycardia*: HR < 40 min-1
- *intervention*: begins with skin preparation, includes surgery and ends with the end of dressing
  - 1. main criterion, making it possible to meet the main objective

Total quantity of remi administered (value related to weight and duration of intervention).

- - 1. secondary criteria to meet secondary objectives:

NB: several criteria are assessed after the patient wakes up, between extubation (H0) and H2 120 min after extubation. The patient does not need to be in the intensive care unit for this assessment; he/she can be in his/her hospital room.

- Proportion of time spent in hemodynamic reactivity, hypotension or bradycardia during the procedure. This proportion will be equal to the cumulative duration of each episode of reactivity, hypotension or bradycardia, divided by the duration of the procedure Total dose of Ephedrine.
- ANIi and ANIm: proportion of time spent during the intervention in the following intervals :

<50, [50-70], >70

- hypnotic component measurement index (BIS® or Entropie®): proportion of time spent during the procedure in the following intervals: <40, [40-60], >60
- ANI, heart rate, blood pressure, FetCO2, Pmax (ventilator) 5 min before start of procedure, 5 min after start of procedure and 20 min after start of procedure.
- Pain assessed by visual analog scale (VAS) between H0 and H2, every 15 min.
- Total quantity of morphine administered between end of surgery and H2 post-op.
- Total quantity of ketamine administered between end of surgery and H2 post-op.
- Frequency of postoperative nausea/vomiting assessed at H2.
- Number of changes in remifentanil flow rate during the procedure.
- Total quantity of propofol administered (absolute value, value related to weight, value related to weight and duration of procedure).
- Number of propofol target changes during the procedure.

In the *"ANI-loop"* arm only: number and total duration of disengagements of the ANI-loop device during the operation, i.e. the number of times and cumulative duration that the closed-loop system had to be interrupted and replaced by manual control, as in the standard practical arm.

##### Calculating the number of subjects

The aim of the study was to assess whether the ANI-loop medical device could reduce the total amount of remifentanil (remi) administered compared with standard practice. Based on literature data [Leal PC et al.] and the department's usual practice, the total amount of remifentanil in the control group can be estimated at 0.30 ± 0.075 (mean ± standard deviation). To demonstrate a 20% reduction in the *ANI-loop* arm (i.e., a mean remifentanil quantity of 0.24 in the experimental group), with a first-species risk of 5% and a power of 80%, we need to include 26 patients per group, for a total of 52 patients.

Reference:Leal PC, Sakata RK, Salomão R, Sadatsune EJ, Issy AM. Braz J Anesthesiol. 2013 Mar- Apr;63(2):178-82.

##### Analysis method and strategy

Statistical analyses will be carried out using SAS software (version 9.4 or higher) at the Lille CHRU's methodological support platform, under the responsibility of Pr A. Duhamel. All statistical tests will be two-sided, with a first-species risk of 5%. No intermediate analyses are planned. A detailed statistical analysis plan will be drawn up and validated before the database is frozen.

Patient characteristics at inclusion will be described for each of the two study arms. Qualitative variables will be described by numbers and percentages. Quantitative variables will be described by the mean and standard deviation in the case of a Gaussian distribution, or by the median and interquartile range (i.e. 25th and 75th percentiles) in the opposite case. The normality of distributions will be tested by a Shapiro-Wilk test and verified graphically by histograms.

Primary objective: The total amount of remifentanil administered (value related to weight and duration of intervention) will be compared between the two arms of the study by a Student's t-test; the effect size will be calculated (standardized mean difference) with a 95% confidence interval. If the distribution of the primary endpoint deviates from normality, the Mann-Whitney U test will be used.

Secondary objectives: Qualitative secondary endpoints will be compared between the two arms of the study using a Chi-square test or Fisher's exact test (when the validity conditions of the Chi-square test are not met). Quantitative secondary endpoints are compared between the two arms of the study using a Student's t-test, or a Mann-Whitney U-test if the distribution of the endpoint deviates from normality. Quantitative secondary endpoints measured at different times will be compared between the two arms of the study using a linear mixed model. This model takes into account the correlation between repeated measurements in the same patient, and the possible existence of missing data. The choice of correlation model will be based on the AIC criterion. The validity of the model will be studied by analyzing the residuals. In the event of significant deviations from normality without obvious transformation of the criterion, the criteria will be compared at each time point between the groups using Mann-Whitney U tests. Finally, secondary criteria identified only in the

The "*ANI-loop*" model will be described using the usual descriptive statistics.

#### Study logistics

##### Participating teams and team experience in the field

Pôle d'Anesthésie Réanimation (Professor Benoît TAVERNIER), CHRU Lille :

The R. Salengro Anesthesia and Intensive Care Clinic is a structure that brings together and coordinates all anesthesia and intensive care activities at the Roger Salengro Hospital. Part of the CHRU's anesthesia-intensive care unit and the University Department of Anesthesia and Emergency Medicine of the Faculty of Medicine, the unit's priority missions are care, innovation, teaching and research in anesthesia-intensive care.

The Clinique R. Salengro comprises six anesthesia-intensive care sectors (emergency, orthopedics-traumatology, "surgical specialties", neurosurgery-neuroradiology, neurosurgical intensive care, burn center). The structure as a whole includes 46 intensive care anesthetists (university-affiliated and hospital-based) and 19 interns, for intensive care anesthesia and perioperative medicine activities organized around 40 operating theaters or

interventional care, 36 SSPI beds, and almost 400 surgical hospital beds, including 18 surgical intensive care beds and 26 surgical intensive care beds.

Team members involved in the research: Dr Mathieu JEANNE (PH), Dr Michel Delecroix (PH), Dr Arnaud ALLUIN (CCU-AH).

INSERM CIC-IT 1403, CHRU Lille:

Certified in January 2008 by INSERM and DHOS, the Clinical Investigation center in Innovative Technologies (CIC-IT) is a functional unit of the Lille CHRU. It has specific national expertise in the "Biosensors and e-Health" theme, focusing on innovation and applications. It is a structure entirely dedicated to clinical research projects involving technological, institutional or industrial innovations, in collaboration with the CIC Plurithématique. The Lille CIC-IT brings together two specialized teams in the field: - the "Biosensors and Instrumentation" team, specialized in the development of new technologies for the acquisition and processing of physiological signals, - the EVALAB laboratory, specialized in the Usability of technological applications in Medicine.

The CIC-IT benefits from the infrastructure of the CHRU de LILLE, and is based at the Maison Régionale de la Recherche Clinique, on the CHRU de Lille campus.

Team members involved in the research: Dr Julien DE JONCKHEERE (research officer), Mathilde FLOCTEIL (clinical research associate), Dr Mathieu JEANNE (PH).

##### Practical aspects of the research

- - 1. Informed consent

It will be obtained after information during the pre-anaesthetic consultation or during the final pre-anaesthetic visit, by one of the investigator(s). The patient will receive the information form and will be asked to sign the consent form in triplicate, after a reasonable period of reflection.

- - 1. Devices used for anesthesia

General anesthesia is induced and maintained by propofol and remifentanil.

- Propofol is administered in both arms by an intravenous anesthesia system with a pK/pD concentration target: Primea Orchestra® base (Fresenius) used routinely in the department.
- Remifentanil is administered
  - in the "*standard practice"* arm, by an intravenous anesthesia system with a concentration target based on a pK/pD model: Primea Orchestra® base (Fresenius) used routinely in the department. Authorized targets according to standard practice range from 0.0 to 10.0 ng.ml^-1^ during anesthesia.
  - In the *ANI-loop* arm, remifentanil is automatically administered by the ANI-loop medical device.
    1. Anesthesia procedure

*Welcoming patients to the operating room*

Installation of standard monitoring equipment

ECG, plethysmography, non-invasive blood pressure (NIBP), entropy, ANI.

Collection of usual hemodynamic constants and calculation of HR thresholds_bl_ and PAS_bl_ used in intra-anaesthesia.

Randomization directly via electronic CRF and allocation to *"standard practice"* or *"ANI-loop"* arm.

The Physiodoloris® monitor (ANI index) remains visible in both study arms.

*Induction of anesthesia*

Similar anesthetic induction in both groups: midazolam 0.08 mg.kg^-1^ IVD

remifentanil target set at 8.0 ng.ml^-1^

propofol target set at 5.0 µg.ml^-1^ then increased in 1 µg.ml increments^-1^ until loss of consciousness.

Tracheal intubation, possibly after curarization

propofol and remifentanil targets lowered to 3 µg.ml^-1^ and 3 ng.ml respectively^-1^

Controlled Assisted Ventilation (CAV): tidal volume of 8 ml.kg^-1^ of ideal weight, ventilatory frequency: 12 cycles.min^-1^

Tidal volume will be adapted to maintain FetCO2 in the range [30 - 35 mmHg].

*Maintenance of anesthesia during the procedure*

- propofol target adapted in both arms to maintain the hypnotic component index (BIS® or Entropy®) in the range [40-60]. During *the procedure*, propofol target changes will be made 1 minute after the last target change has become effective, and will follow the indications in table 1. This corresponds to the usual management in the burn unit of the Burn Treatment Center.

|  |  |  |
| --- | --- | --- |
| component  hypnotic | propofol target |  |
| >80 | + 2.0 |  |
| >60 | + 1.0 |  |
| <40 | - 0.5 |  |
| <25 | - 1.0 |  |
| **Table 1: Adaptation of propofol targets in both arms** | | |

|  |  |  |
| --- | --- | --- |
| ANI | target remi |  |
| <35 | + 2.0 |  |
| <50 | + 1.0 |  |
| >70 | - 0.5 |  |
| >90 | - 1.0 |  |
| **Table 2: Adaptation of remi targets in the "standard practice" arm** | | |

- remifentanil target
  - **in the *standard practice* arm**: increased to 4 ng.ml-1 at the start of *the procedure*, then adjusted to maintain ANI in the range [50-70] as indicated in Table 2. Target changes are made 1 minute after the last target change has become effective. This corresponds to usual management at the Burn Treatment Center.
  - **in the *ANI-loop* arm**, the automatic mode of the ANI-loop device is engaged at the start of *the procedure*. The flow of remifentanil is automatically regulated by the ANI-loop. The anaesthetist can switch to manual mode at any time in the event of inadequate regulation (device disengagement). The device is switched off at the end of the *procedure.*

Pre-emptive analgesia

- local infiltration of skin sample area with naropein 2 mg.ml-1 during

*intervention*

- paracetamol 1g IVL at end of *procedure*
- sufentanil 0.12 µg.kg-1 (max 10 µg) IVL at *end of procedure*

Prevention of postoperative nausea/vomiting

- ondansetron 4 mg IVL

Postoperative analgesia

- morphine titration according to Visual Analogue Scale (VAS), bolus of 3mg every 3 min (max 12mg)
- ketamine 20 mg IVL for persistent pain with VAS ≥ 40

##### Duration

The sponsor reserves the right to discontinue the trial in the event of non-inclusion. The study may be stopped by a joint decision of the competent authority, the sponsor or the principal investigator.

- duration of inclusion period: 12 months
- duration of study participation for one subject: from arrival in the OR to H2 post-op.
- duration of research: 15 months
- data analysis time: 3 months

##### Criteria for discontinuing study participation

Each subject may leave the study by decision of the competent administrative authority, the sponsor and the principal investigator, but also by decision of an investigator or by decision of the subject him/herself, in accordance with the regulations and as mentioned in the consent form.

- the presence of a major extrasystole (more than two extrasystoles per minute without interruption) is a criterion for stopping a patient's participation in the study, because ANI measurements are not possible under these conditions.
- In this case, the patient's inclusion would be deemed null and void; the investigating center would then proceed with an additional inclusion in replacement.
- no follow-up of the excluded patient is justified by participation in the study.

##### Ban on simultaneous participation - Exclusion period

Simultaneous participation in another study is prohibited because it would run the risk of interacting with autonomic nervous system responses. This prohibition is limited to the duration of the subject's participation in the study.

##### Study benefits, risks and constraints

- - 1. PROFIT

Use of the device should result in more stable hemodynamics and therefore better anesthesia quality, as well as potentially lower total remi administration.

- - 1. COLLECTIVE PROFIT

Better quality anesthesia would probably benefit the overall quality of perioperative care in the long term.

- - 1. RISKS
- standard practice" arm: the risk is identical to that of any general anaesthetic
- *ANI-loop"* arm: in the event of failure of the ANI-loop device, the anesthetist can disengage it at any time and continue anesthetic management according to "standard practice" without delay. The risk is therefore comparable to that of the "standard practice" arm.
  - 1. Supervisory Board
- in view of the similar risk in the *"ANI-loop"* arm and in the "standard practice" arm, the setting up of a monitoring committee does not seem justified.

#### Medical devices

See appendice II.

#### Safety assessment

##### Definition

- - 1. Undesirable event

Any harmful event occurring in a person undergoing research involving the human body, whether or not the event is related to the research or the product to which the research relates.

- - 1. Undesirable effect

Any adverse event related to the research or the product to which the research relates.

- - 1. Serious adverse event or reaction

Any event or adverse reaction that :

- leads to death,
- endangers the life of the person undergoing the research,
- requires hospitalization or prolongation of hospitalization,
- causes significant or lasting disability or handicap,
- or results in a congenital anomaly or malformation, regardless of the dose administered.
- is deemed medically serious by the investigator.

Certain circumstances requiring hospitalization do not fall under the severity criterion.

"hospitalization or prolongation of hospitalization" as :

- admission for social or administrative reasons
- protocol-defined hospitalization
- hospitalization for medical or surgical treatment scheduled prior to research
- transfer to day hospital
  - 1. Unexpected adverse reaction

Any undesirable effect whose nature, severity or course is not consistent with the information on the products, procedures and methods used in the research.

- - 1. New fact

Any new data that may lead to a reassessment of the risk-benefit ratio of the research or of the investigational product, to changes in the use of this product, in the conduct of the research, or in the documents relating to the research, or to the suspension, interruption or modification of the research protocol or similar research.

##### Description of safety assessment parameters and protocol risks

- standard practice" arm: the risk is identical to that of any general anaesthetic
- *ANI-loop"* arm: in the event of failure of the ANI-loop device, the anesthetist can disengage it at any time and continue anesthetic management according to "standard practice" without delay. The risk is therefore comparable to that of the "standard practice" arm.

##### Adverse events related to Propofol and Remifentanil

- - 1. Propofol-related adverse events

Induction and maintenance of anesthesia or sedation with propofol are generally easy, with minimal signs of excitation. The most frequently reported adverse events with propofol are those pharmacologically predictable for an anesthetic/sedative agent, such as hypotension. The nature, severity and incidence of adverse events observed in patients receiving propofol may be related to the patient's condition and the operative or therapeutic procedures implemented:

Table of adverse drug reactions

| **Class of organ system** | **Frequency** | **Undesirable effects** |
| --- | --- | --- |
| **Conditions of immune system :** | *Very rare*  (<1/10 000) | Anaphylaxis - can include angioedema, bronchospasm, erythema  and hypotension |
| **Metabolism and nutrition disorders:** | *Undetermined frequency*  (9) | Metabolic acidosis^(5)^ , hyperkalemia  ^(5)^hyperlipidemia ^(5)^ |
| **Psychiatric disorders :** | *Undetermined frequency*  (9) | Mood euphoric, abuse and drug dependence  (8) |
| **Conditions of the Nervous system :** | *Common*  (>1/100, <1/10) | Headaches during the waking phase |
|  | *Rare*  (>1/10 000, <1/1 000) | Epileptiform movements, including convulsions and opisthotonos during induction, maintenance and recovery |
|  | *Very rare*  (<1/10 000) | Postoperative unconsciousness |
|  | *Undetermined frequency*  (9) | Involuntary movements |
| **Cardiac disorders :** | *Common*  (>1/100, <1/10) | Bradycardia ^(1)^ |
|  | *Very rare*  (<1/10 000) | Pulmonary edema |
|  | *Undetermined frequency*  (9) | Arrhythmia cardiac ^(5)^, heart failure^(5)^ , ^(7)^ |
| **Vascular disorders :** | *Common*  (>1/100, <1/10) | Hypotension ^(2)^ |
|  | *Uncommon*  (>1/1 000, <1/100) | Thrombosis and phlebitis at the injection site |
| **Conditions respiratory, thoracic and mediastinal :** | *Common*  (>1/100, <1/10) | Transient apnea during induction |
|  | *Undetermined frequency*  (9) | Depression respiratory (dose - dependent) |
| **Gastrointestinal disorders:** | *Common*  (>1/100, <1/10) | Nausea and vomiting during the awakening phase |
|  | *Very rare*  (<1/10 000) | Pancreatitis |
| **Hepatobiliary disorders :** | *Undetermined frequency*  (9) | Hepatomegaly ^(5)^ |
| **Conditions musculoskeletal and systemic:** | *Undetermined frequency*  (9) | Rhabdomyolysis^(3)^ , ^(5)^ |
| **Kidney and urinary tract disorders :** | *Very rare*  (<1/10 000) | Decolorization from urine after prolonged administration |
|  | *Undetermined frequency*  (9) | Renal insufficiency ^(5)^ |

| **Class of organ system** | **Frequency** | **Undesirable effects** |
| --- | --- | --- |
| **Reproductive organs and breast disorders :** | *Very rare*  (<1/10 000) | Sexual disinhibition |
| **General disorders and abnormalities at administration site :** | *Very common*  (>1/10) | Local pain on induction ^(4)^ |
|  | *Very rare*  (<1/10 000) | Tissue necrosis^(10)^ 'following accidental extravascular administration administration |
|  | *Undetermined frequency*  (9) | Local pain, oedema, following a  administration accidental extravascular |
| **Investigations :** | *Undetermined frequency*  (9) | Brugada ECG^(5)^ , ^(6)^ |
| **Lesions, intoxications and complications linked à**  **intervention :** | *Very rare*  (<1/10 000) | Postoperative fever |

^(1)^ Severe bradycardia is rare. Isolated cases of evolution towards asystole have been reported.

^(2)^ Hypotension may occasionally require the use of intravenous fluids and a reduction in the rate of propofol administration.

^(3)^ Very rare cases of rhabdomyolysis have been reported when propofol was administered at doses higher than 4 mg/kg/h for sedation in intensive care units.

^(4)^ Local pain can be minimized by using larger veins in the forearm and antecubital fossa. With PROPOFOL LIPURO 10 mg/ml, local pain can also be minimized by co-administration of lidocaine.

^(5)^ Combinations of these events, known as "propofol infusion syndrome", may be observed in critically ill patients who often have multiple risk factors for the development of events, see section 4.4.

^(6)^ Brugada ECG - ST-segment elevation with domed appearance and negative T wave on ECG.

^(7)^ Rapidly progressive heart failure (with fatal outcome in some cases) in adults. In such cases, heart failure generally does not respond to supportive inotropic therapy.

^(8)^ Drug abuse and dependence on propofol, most often by healthcare professionals.

^(9)^ Undetermined frequency (cannot be estimated on the basis of available data).

^(10)^ Necrosis has been reported where tissue damage has occurred.

- - 1. Adverse events related to Remifentanil

The adverse effects most frequently observed with remifentanil are directly related to the pharmacology of morphine agonists. These side effects disappear within minutes of stopping or reducing the rate of remifentanil administration.

The adverse reaction frequencies below are defined as: very common (≥ 1/10), common (≥ 1 /100 and < 1/10), uncommon (≥ 1/1,000 and < 1/100), rare (≥ 1/10,000 and < 1/1,000) and very rare (< 1/10,000).

| **Immune system disorders** | |
| --- | --- |
| Rare | Allergic reactions, including anaphylactic reactions, have been reported in patients receiving remifentanil in combination with one or more anesthetic agents. |
| **Nervous system disorders** | |
| Very frequent | Rigidity of skeletal muscles |
| Rare | Sedation (during the awakening phase) |

| Cardiac disorders | |
| --- | --- |
| Frequent | Bradycardia |
| Rare | Asystole/cardiac arrest, usually preceded by bradycardia, has been reported in patients with  received remifentanil in combination with other anesthetic agents. |
| **Vascular disorders** | |
| Very frequent | Hypotension |
| Frequent | Postoperative hypertension |
| **Respiratory, thoracic and mediastinal disorders** | |
| Frequent | Acute respiratory depression, apnea |
| Infrequent | Hypoxia |
| **Gastrointestinal disorders** | |
| Very frequent | Nausea, vomiting |
| Infrequent | Constipation |
| **Skin and subcutaneous tissue disorders** | |
| Frequent | Pruritus |
| **General disorders and administration site abnormalities** | |
| Frequent | Postoperative chills |
| Infrequent | Postoperative pain |

##### Procedures for recording and reporting adverse events

- - 1. Investigator's responsibilities

Collection of adverse events

All adverse events will be reported on the adverse event forms in the observation book. Each adverse event observed will be recorded individually. The intensity of adverse events will be determined as follows

- - mild (grade 1): no interference with the patient's daily activity;
  - moderate (grade 2): moderate interference with the patient's daily activity, but still acceptable;
  - severe (grade 3): significant interference with the patient's daily activities and unacceptable ;
  - life-threatening (grade 4);
  - death (grade 5).

All adverse events must be graded and evaluated. Notification of serious adverse events

The investigator must notify the sponsor of all serious adverse events occurring during the trial period, with the exception of those listed in the protocol as not requiring notification, without delay from the date of knowledge.

All serious undesirable events must be reported on a "Serious Undesirable Event" form in the observation book.

This form should be sent to the promoter (Cellule Vigilance de la Fédération de Recherche Clinique) by fax to 03 20 44 57 11.

For each undesirable event, the investigator must document the event to the best of his or her ability:

- a clear, detailed description of the event, in the form of a medical diagnosis if possible
- the severity, start and end dates of the event, and its evolution
- the causal link between the serious undesirable event and the medical device or the procedure used to implement it.

Adverse events will be monitored by the investigator.

For each SAE, the investigator must provide the following information, anonymously and whenever possible:

- a copy of the hospitalization or extended hospitalization report
- a copy of all relevant additional test results
- any other document it deems useful and relevant

Reporting period for serious adverse events

If a research participant is involved in an SAE, it must be reported:

- - from the date consent is signed,
  - for the duration of the trial,
  - and until the end of the subject's participation in the trial,
  - without time limit, when it is likely to be due to the research / experimental drug(s) / device(s) tested (e.g. serious effects that may appear at a great distance from exposure to the drug, such as cancer or congenital anomalies).

Pregnancy reporting

Pregnancy does not constitute a serious adverse event, but its occurrence during the trial must be notified without delay, on the standard pregnancy reporting form, to the sponsor, who will ensure that it runs smoothly if deemed necessary.

The investigator must follow the patient until the end of the pregnancy or its termination, and notify the sponsor of the outcome using the standard pregnancy outcome form.

If the outcome of the pregnancy falls within the definition of serious adverse events (spontaneous abortion with hospitalization, fetal death, congenital anomaly, etc.), the investigator must follow the procedure for reporting serious adverse events.

- - 1. The promoter's responsibilities

Reporting serious and unexpected adverse reactions

For each serious adverse event or reaction, the sponsor assesses the severity and causal link between the event or reaction and the study product(s) or protocol, as well as its unexpectedness.

The sponsor notifies the ANSM and the CPP of any **suspected serious and unexpected adverse reaction**:

- in the case of **an unexpected serious adverse reaction resulting in death or life-threatening** illness, without delay from the day on which the sponsor becomes aware of the event;
- in the case of **other serious unexpected adverse reactions**, no later than fifteen days after the day on which the sponsor became aware of them.

The sponsor declares, in the form of a follow-up report to the ANSM and the CPP, additional relevant information concerning :

- suspected life-threatening or fatal unexpected serious adverse reactions, within eight days of the day on which the sponsor becomes aware of them.
- other cases of suspected serious unexpected adverse reactions, within a further period of eight days from the fifteen-day period for the initial report.

Declaration of safety developments

In the event of a new safety event occurring during the course of the study, the sponsor will immediately notify the CPP and ANSM by e-mail, as soon as it becomes aware of the new event and any measures taken.

Annual safety report

Once a year for the duration of the trial, or on request, the sponsor submits a **safety report** to the ANSM and the CPP**.** This safety report will include an overall analysis of the safety profile of the study protocol, taking into account all relevant new safety data. Safety information will appear in the form of summary tables summarizing serious adverse events or reactions that have occurred in biomedical research.

#### Right of access to source data and documents

The investigator undertakes to accept controls by the sponsor (monitor and/or auditor), or by the inspector of the competent administrative authority. He/she guarantees access to source data (medical records, computer files, study documents, etc.).

#### Quality control and assurance

The Quality Assurance approach that will be implemented means that research subjects can be cared for under the best possible conditions in terms of safety and compliance with medical and regulatory rules.

- Test sequence

Medical observations will be kept in the patient's file, and data relating to the study will be recorded in the observation notebooks provided for the study, in accordance with good clinical practice, covering the various stages of patient management in the protocol. Any deviation from the protocol will be reported, together with the reason for it. Data collection must be exhaustive, and will be regularly checked by a Clinical Research Assistant in accordance with protocol procedures (if applicable).

- Study monitoring

Monitoring of the trial will be carried out according to the monitoring schedule validated before the start of the study, or on specific request by the sponsor's CRA. It will depend on the number of patients included in the study.

A set-up meeting with the principal investigator will be held before the start of the trial (reminder of GCP, research organization, planned monitoring).

The investigator informs the sponsor in real time of the inclusions made.

During on-site monitoring visits, ARCs should be able to consult :

- data collection notebooks for included patients
- patient medical and nursing records
- investigator's binder

The monitoring will check at least the following 6 points:

- the existence of patients, information and the presence of signed informed consents
- compliance with inclusion criteria
- primary endpoint
- monitoring and reporting of SAEs
- new facts requiring the tabling of an amendment
- Management and control of medical devices
- Closing the study

At the end of the trial, closing procedures will be applied, with the filing of all documents and source data. Once the final analysis has been carried out and validated, all files and data are sealed and archived according to specific procedures in secure premises.

#### Ethical and legal considerations

The trial will be conducted in accordance with the approved protocol, in compliance with the French Public Health Code, the EU GCP and applicable regulatory requirements. The trial will be registered on the public database ClinicalTrials.gov.

##### Personal Data Protection Committee and Competent Authority

- - 1. Favourable opinion of the CPP

The sponsor submits a request for an opinion to the CPP before the research begins, in accordance with article L1121-4 of the French Public Health Code. The sponsor sends a copy and a summary of the research to ANSM.

- - 1. Changes to the protocol

The sponsor alone is authorized to modify the protocol, in consultation with the principal investigator.

Substantial modifications are those which have a significant impact on any aspect of the research, in particular on the protection of persons, including their safety, on the conditions of validity of the research, where applicable on the quality and safety of the products tested, on the interpretation of scientific documents supporting the conduct of the research, or on the methods of conducting the research.

A request for substantial modification is sent by the sponsor to the CPP. On receipt of a favorable opinion, the amended version of the protocol is then sent by the sponsor to the ANSM for information, and to all investigators.

A non-substantial amendment to the protocol is a minor modification or clarification that has no impact on the conduct of the trial. These modifications will not be submitted to the competent authorities, but will be agreed between the sponsor and the investigator and clearly documented (in the study follow-up file).

##### Information and consent

In accordance with current regulations, trial participants must receive fair and complete information in the form of an Information Letter, specially drafted for the trial and validated by the French Comité de Protection des Personnes, which must be provided and explained by the Investigator. In particular, the Investigator must inform the participant of the possible risks and constraints of participating in the trial.

Participants will be able to ask any questions they may have, and will be given the time they need to make an informed decision.

The trial participant must then sign the consent form with the Investigator who presented the trial, and this document must be dated on the day of signature. One copy will be given to the participant, one will be kept by the Investigator, and one will be kept in the participant's medical file.

The Investigator must ensure that the inclusion and exclusion criteria are met before the participant is included. No specific research procedure may be carried out before the participant has been informed and given consent to take part in the trial. Participants may withdraw their consent at any time, and all information concerning the safety of their participation must be reported to them.

Registration in the national database of persons undergoing biomedical research

People who take part in biomedical research are not registered in the national database, because the study is related to the pathological condition, and they do not receive any compensation.

#### Data processing and storage of documents and research data

Data processing will be carried out under the conditions of confidentiality defined by the French Data Protection Act of January 6, 1978 (CNIL). Data processing will be carried out in accordance with the requirements of CNIL reference methodology MR 06001.

In order to best meet the objectives of this study, all the variables will be drawn up in collaboration with the investigator, the datamanager and the biostatisticians.

The paper CRF will then be formatted to organize the data and enable logical collection according to the patient pathway or location of the data to be collected.

The final version of the CRF will be validated by the investigator, the datamanager and the biostatisticians.

This final version will be used to develop an eCRF by Pr Duhamel's Unité de Méthodologie, Biostatistiques et Datamanagement at the Lille CHRU.

The eCRF will be developed on Ennov Clinical, which is FDA-approved and offers optimal database quality.

This eCRF will enable on-the-fly tests to be carried out in relation to the expected data dictionary, as well as data consistency tests. These tests will be defined by the investigator and the data manager prior to the development of the input mask.

Final data monitoring, defined by the consistency tests set up, will be carried out to check the consistency and quality of the data entered.

Data will be collected directly on the eCRF. Patient anonymization will be managed outside the eCRF.

The center must hold the list of patient identifier/identity correspondences in a secure manner that is inaccessible to people who do not have consultation rights. These identifiers will be transferred to the eCRF.

Data will be hosted at the CHRU in Lille, via our partner eSIS (Groupement d'Intérêt Public named GIP e-SiS 59/62).

Data concerning this study will be archived for a minimum period of fifteen years from the end of the research or its early termination, without prejudice to the legislative and regulatory provisions in force.

#### Financing and insurance

##### Financing

The medical devices are supplied free of charge by MetroDoloris®.

##### Insurance

The sponsor has taken out an insurance policy covering its civil liability and that of all participants in the study, in accordance with article L1121-10 of the French Public Health Code.

#### Publication- Valuation

In accordance with article R 5121-13 of the French Public Health Code, no written or oral comment may be made on trials without the joint agreement of the investigator and the sponsor. All publications must mention that the CHRU de Lille is the sponsor (identification number "2010_51" to be requested from the Health Research Department). In all cases, the CHRU de Lille, as study promoter, is responsible for the first publication. The investigator sends a copy of his publications to the promoter.

The sponsor is the sole owner of the study results. Under no circumstances may these results, or any other data relating to the research, be passed on to a third party without prior negotiation with the Health Research Department. Any such request must be forwarded as soon as possible to the legal affairs department of the Département de la Recherche en Santé.

#### List of appendices

**Appendix I**: ANI-based anesthesia regulation: description of the ANI-LOOP device

**Appendix II**: State of the art in loop delivery systems for intravenous anesthetics

### Appendix I: ANI-based anesthesia regulation: description of the ANI-LOOP device

*ANI computation*

The ECG is digitized at a sampling rate of 250 Hz. ECG R waves are then detected in order to build the RR intervals series defined as the time evolution of the time intervals between two R waves. RR series is analyzed using an original non linear filtering algorithm in order to detect and replace each disturbed RR sample. Filtered RR series are then re-sampled at 8 Hz using a linear interpolation. RR series is then mean centered and normalized into a 64 seconds moving window. Since the method is based on the analysis of HF changes, the RR series is band pass filtered between [0.15-0.4 Hz]. The band pass filtering is realized using a numerical filter based on the 4 coefficients Daubechies wavelet. Local maxima and minima are detected and the upper and lower envelopes are plotted by connecting the local minima together and the local maxima as well (red curves). The 64 sec moving window is then divided into four sub-windows of 16 sec. The areas between the lower and upper envelopes are then measured in the four sub-windows. We defined AUCmin as the smallest of these sub-areas.

ANI is then computed in order to obtain a value between 0 and 100:

ANI = 100 * [a*AUCmin+b] / 12.8 (1)

Where *a* = 5.1 and *b* = 1.2 have been empirically determined in a data set of more than 100 anesthetized patients in order to obtain a good correlation between the visual pattern of the parasympathetic influence on RR series and the quantitative measurement of ANI.

This parameter is available on the Physiodoloris® monitor commercialised by MetroDoloris® (Lille, France). The monitor user interface displays in real time the instantaneous and averaged values of the ANI index (respectively ANI_i_ in yellow and ANI_a_ in orange). The different index values are also available in real time through the classical RS-232 monitor serial port.

*B - Decision rules for analgesic drugs administration*

Since 2008, the Physiodoloris® monitor has been used in the orthopedic surgery unit of the Lille University hospital. We observed and recorded ANI evolution and reactions on more than 2000 patients during orthopedic surgical procedure under general anesthesia. Thanks to this experience, we determined several decision rules based on ANI_i_ and ANI_a_ analysis in order to help anesthesiologists understand ANI guided anesthesia. In order to regulate analgesic drug administration, we determined two types of action:

- ***Infusion flow Changes:*** Increase or decrease the syringe pump infusion rate.
- ***Bolus:*** Infusion rate fast increase during 10 s. Infusion rate returns to its previous value after these 10s.

Each action is followed by a *refractory period* which shuts down the regulation algorithm for several seconds.

Several regulation variables and constants are defined.

*Variables used for regulation:*

- ***ANI_i_ :*** instantaneous ANI.
- ***ANI_a_ :*** average ANI.
- ***S_i_*** : ANI_i_ slope (computed on 30 s).
- ***S_a_*** : ANI_a_ slope (computed on 30 s).
- ***InF:*** Syringe pump infusion Flow (in µg/Kg/min).

*Constants used for regulation (set by the user):*

- ***ANI_max_*** : representing the ANI max value.
- ***ANI_min_*** : representing the ANI min value.
- ***S_a_*** T: representing the S_a_ threshold
- ***S_i_*** T: representing the S_i_ threshold
- ***InI:*** Infusion Rate increment (in µg/Kg/min).
- ***InF_min_*** : Minimum infusion rate (in µg/Kg/min).
- ***Inf_max_*** : Maximum infusion rate (in µg/Kg/min).
- ***BolusI:*** Infusion Flow bolus increment (in µg/Kg/min).
- ***RefP****:* Refractory period (in s)

***ANI_mean_*** is computed as

ANI_mean_ = ANI_min_ +(ANI_max_ -ANI_min_ )/2 (2)

The defined rules are established in order to treat both acute pain and global analgesia level changes.

*Acute pain treatment:*

**If**(ANI_i_ < ANI_min_ **)and(**S_i_ <-S_i_ T)**then** Bolus (3) Bolus corresponds to an Infusion rate increase of *Bolus* µg/Kg/min during 10 s.

Infusion rate returns to its previous baseline after these 10s.

Each Bolus is followed by a 3*RefP refractory period.

*Analgesia level changes:*

#### ANI_a_ <ANI :_min_

In this area, we consider that analgesia is insufficient. This event is treated by increasing the infusion pump rate.

**If(**InF<InFmax)**and**(S_a_ <SaT) **then** InF = InF+InI (4)

#### ANI_min_ <ANIa< ANI_max_ :

In this area, we consider that analgesia in adequate. However, in order to anticipate, we consider the ANI_a_ slope (S_a_ ) changes in order to regulate the infusion rate.

**If**(ANI_a_ <ANI_mean_ **)and**(S_a_ <-S_a_ T)**and**(InF+InI<InF )_max_

**then** InF = InF+ InI (5)

**If**(ANI_a_ > ANI_mean_ **)and** (S_a_ >S_a_ T)**and**(InF- InI>InF )_min_

#### ANIa>75 :

**then** InF = InF- InI (6)

In certain condition, we consider that if ANI is too high, ANS doesn't respond to noxious stimuli. This effect could be explained by an opioid overdose which would require an infusion flow decrease.

**If**(S_a_ >0)**and**(InF- InI>InF_min_ **)then** InF = InF- InI (7) Each infusion rate change is followed by a RefP refractory period.

In order to avoid arterial hypotension (SBP<80 mmHg), we added two rules on systolic blood pressure (SBP).

**If** SBP<90 **then** InF = InFmin (8)

**If** SBP<80 **then** InF = 0 (9)

In the case of SBP<80 mmHg, the regulation algorithm is shut down until SBP recovers a value over 85 mmHg. Rules (8) and (9) have the highest level of priority.

*C - Algorithm implementation*

From a technical point of view, the system consists in a software ("controller") implemented on a classical personal computer allowing to adapt in real time the syringe pump infusion rate according to ANI_i_ and ANI_a_ evolutions. ANI_i_ and ANI_a_ values are obtained from the Physiodoloris monitor communication interface and SBP is obtained from the Anesthesia monitor (Datex Ohmeda AS-5, GE Healthcare or

Intelliview® Philips^TM^ ). The syringe pump (Alaris GH, Cardinal health) and the two monitors are linked to the computer through a Keyspan® 4-port serial to USB adapter.

The software also allows users to enter patient characteristics (age, size, weight, ASA) and constants values used for regulation. Thanks to the interactive aspect of the controller, all the predefined constants can be adapted and manually changed during the regulation. Finally, a specific software interface allows users to follow the variable's evolution, the syringe pump status and the regulation results. Direct syringe pump commands are also available in order to allow the anesthesiologists to manually adapt the syringe pump infusion flow or to shut down the automatic regulation.

Constants used for regulation are defined as follow:

- ***ANI =75_max_***
- ***ANI =50_min_***
- ***S T=7_a_***
- ***S T=25_i_***
- ***InI=0.01 µg/kg/min***
- ***InF_min_ =0.05 µg/kg/min***
- ***Inf_max_ =0.6 µg/kg/min***
- ***BolusI= 0.04 µg/kg/min***
- ***RefP=10 s***

Blood pressure is measured every 2.5 minutes.

#### APPENDIX II: State of the art in loop delivery systems for intravenous anesthetics

The CE-marked PhysioDoloris monitor (MDoloris Medical Systems, Loos, France), on the market since 2010, is a recognized measure of the state of the autonomic nervous system, enabling real-time assessment of parasympathetic tone (paraS) based on non-invasive electrocardiogram collection. It continuously displays the Analgesia Nociception Index (ANI), corresponding to the relative amount of paraS tone, which varies between 0 and 100. Several studies have demonstrated that ANI provides a measure of the state of the antinociception/nociception balance during general anesthesia^8,9,11-14^ . In the conscious patient in the post-interventional monitoring room, after general anaesthesia, ANI enables pain levels to be measured^15^ , except after prolonged sevoflurane anaesthesia^16^ . Outside the perioperative period, in the conscious patient, ANI shows a significant relationship with pain levels, in both children^17^ and adults .^18^

#### It is important to note that the proposed study does not concern the PhysioDoloris monitor. The study focuses on an innovative medical device (see appendix II) which enables the automated administration of remifentanil based on elements from the usual hemodynamic monitoring (heart rate and blood pressure) combined with the continuous measurement provided by the PhysioDoloris monitor (ANI).

Automated administration of propofol for induction and maintenance of general anesthesia (GA) is a technique used by many teams today. The target signal to be maintained within a predefined interval is derived from the electroencephalogram (EEG) in the vast majority of cases: it is the BiSpectral® index (Aspect Medical Systems, MA) and EEG entropy (GE HealthCare). Two medical devices, EasyTIVA® (MedSteer, Suresnes, France) and McSleepy® (McGill University, Quebec, Canada), are currently in the CE marking phase, having completed pre-industrial proof of concept^19,20^ . A third medical device for closed-loop administration of total intravenous general anesthesia (TIVA) has been CE-marked and marketed since 2016, the Concert-CL® (Veryark® Technology Co., Ltd., Guangxi, China) .^21,22^

The analgesic component of GA has seen significant progress over the last decade, with the commercialization of several autonomic nervous system (ANS) monitors that have been used to measure the anti-nociception/nociception balance during GA^23-31^ . The absence of a specific physiological signal for nociception is the main technical difficulty in developing a feedback loop for the administration of morphine analgesia. Some authors have demonstrated that mean arterial pressure can be used as a target for closed-loop administration of alfentanil^32^ , while others have developed a specific score, the analgoscore, which combines variations in heart rate and arterial pressure (McSleepy® system)^19,20^ . Finally, other authors use EEG and cortical arousal induced by nociceptive phenomena to distinguish between the patient's need for hypnotics and morphine (EasyTIVA® system): this device has already been extensively evaluated in a variety of clinical settings^33,34^ , and has demonstrated a real advantage in assessing the morphine-sparing effects of dexmedetomidine use^35^ or the morphine needs of special populations such as morbidly obese patients^36^ . Two recent meta-analyses highlight the safe use of closed-loop delivery devices, and in some cases their superiority to manual delivery systems .^37,38^

**Bibliography**

1. Aubrun F, Nouette Gaulain K, Fletcher D, Belbachir A, Beloeil H, Carles M, Cuvillon P, Dadure C, Lebuffe G, Marret E, Martinez V, Olivier M, Sabourdin N, Zetlaoui P: Réactualisation de la recommandation sur la douleur postopératoire. Revision of expert panel guidelines on postoperative pain management. Anesth Réanim 2016; 2: 421-30
2. Pellat JM, Hodaj H, Alibeu JP, Payen JF, Jacquot C: Postoperative hyperalgesia Clinical description, mechanisms and prevention. Douleurs 2006; 7: 11-16
3. Richebe P, Pouquet O, Jelacic S, Mehta S, Calderon J, Picard W, Rivat C, Alex Cahana A, Janvier G: Target-Controlled Dosing of Remifentanil During Cardiac Surgery Reduces Postoperative Hyperalgesia. J Cardiothorac Vasc Anesth 2011; 25: 917-25
4. Task_Force: Heart rate variability. Standards of measurement, physiological interpretation and clinical use. Task Force of the European Society of Cardiology and the North American Society of Pacing and Electrophysiology. Circulation 1996; 93: 1043-65
5. Jeanne M, Logier R, De Jonckheere J, Tavernier B: Heart rate variability during total intravenous anesthesia: effects of nociception and analgesia. Auton Neurosci 2009; 147: 91-6
6. Jeanne M, Logier R, De Jonckheere J, Tavernier B: Validation of a graphic measurement of heart rate variability to assess analgesia/nociception balance during general anesthesia. Conf Proc IEEE Eng Med Biol Soc 2009; 1: 1840-3
7. Jeanne M, Clement C, De Jonckheere J, Logier R, Tavernier B: Variations of the analgesia nociception index during general anaesthesia for laparoscopic abdominal surgery. J Clin Monit Comput 2012; 26: 289-94
8. Gruenewald M, Ilies C, Herz J, Schoenherr T, Fudickar A, Höcker J, Bein B: Influence of nociceptive stimulation on analgesia nociception index (ANI) during propofol-remifentanil anaesthesia. Br J Anaesth 2013; [Epub ahead of print]
9. Sabourdin N, Arnaout M, Louvet N, Guye ML, Piana F, Constant I: Pain monitoring in anesthetized children: first assessment of skin conductance and analgesia-nociception index at different infusion rates of remifentanil. Paediatr Anaesth 2013; 23: 149-55
10. Jeanne M, de jonckheere J, Butruille L, Logier R, Tavernier B: Estimating the analgesia/nociception balance with the ANI index. OXYMAG 2015; 140
11. Migeon A, Desgranges FP, Chassard D, Blaise BJ, De Queiroz M, Stewart A, Cejka JC, Combet S, Rhondali O: Pupillary reflex dilatation and analgesia nociception index monitoring to assess the effectiveness of regional anesthesia in children anesthetized with sevoflurane. Paediatr Anaesth 2013; 23: 1160-5
12. Boselli E, Logier R, Bouvet L, Allaouchiche B: Prediction of hemodynamic reactivity using dynamic variations of Analgesia / Nociception Index (dANI). J Clin Monit Comput 2015; [Epub ahead of print]
13. Boselli E, Bouvet L, Bégou G, Torkmani S, Allaouchiche B: Prediction of haemodynamic reactivity during total intravenous anaesthesia for suspension laryngoscopy using Analgesia/Nociception Index (ANI): a prospective observational study. Minerva Anestesiol 2015; 81: 288-97
14. Boselli E, Logier R, Bouvet L, Allaouchiche B: Prediction of hemodynamic reactivity using dynamic variations of Analgesia/Nociception Index (ANI). J Clin Monit Comput 2016; 30: 977-984
15. Boselli E, Daniela-Ionescu M, Bégou G, Bouvet L, Dabouz R, Magnin C, Allaouchiche B: Prospective observational study of the non-invasive assessment of immediate postoperative pain using the analgesia/nociception index (ANI). Br J Anaesth 2013; 111: 453-9
16. Ledowski T, Tiong WS, Lee C, Wong B, Fiori T, Parker N: Analgesia nociception index: evaluation as a new parameter for acute postoperative pain. Br J Anaesth 2013; 111: 627-9
17. Avez-Couturier J, De Jonckheere J, Jeanne M, Vallée L, Cuisset JM, Logier R: Assessment of Procedural Pain in Children Using Analgesia Nociception Index: A Pilot Study. clin J Pain 2016
18. Le Guen M, Jeanne M, Sievert K, Al Moubarik M, Chazot T, Laloë PA, Dreyfus JF, Fischler M: The Analgesia Nociception Index: a pilot study to evaluation of a new pain parameter during labor. Int J Obstet Anesth 2012; 21: 146-51
19. Charabati S, Bracco D, Mathieu PA, Hemmerling TM: Comparison of four different display designs of a novel anaesthetic monitoring system, the 'integrated monitor of anaesthesia (IMA)'. Br J Anaesth 2009; 103: 670-7
20. Hemmerling TM, Arbeid E, Wehbe M, Cyr S, Taddei R, Zaouter C: Evaluation of a novel closed-loop total intravenous anaesthesia drug delivery system: a randomized controlled trial. Br J Anaesth 2013; 110: 1031-9
21. Liu Y, Li M, Yang D, Zhang X, Wu A, Yao S, Xue Z, Yue Y: Closed-loop control better than open-loop control of profofol TCI guided by BIS: a randomized, controlled, multicenter clinical trial to evaluate the CONCERT-CL closed-loop system. PLoS ONE 2015; 10
22. Liu Y, Li M, Yang D, Zhang X, Wu A, Yao S, Xue Z, Yue Y: Closed-loop control better than open-loop control of profofol TCI guided by BIS: a randomized, controlled, multicenter clinical trial to evaluate the CONCERT-CL closed-loop system. PLoS ONE 2015; 10
23. Struys MM, Vanpeteghem C, Huiku M, Uutela K, Blyaert NB, Mortier EP: Changes in a surgical stress index in response to standardized pain stimuli during propofol-remifentanil infusion. Br J Anaesth 2007; 99: 359-67
24. Wennervirta J, Hynynen M, Koivusalo AM, Uutela K, Huiku M, Vakkuri A: Surgical stress index as a measure of nociception/antinociception balance during general anesthesia. Acta Anaesthesiol Scand 2008; 52: 1038-45
25. Bonhomme V, Uutela K, Hans G, Maquoi I, Born JD, Brichant JF, Lamy M, Hans P: Comparison of the Surgical Pleth IndexTM with haemodynamic variables to assess nociception-anti-nociception balance during general anaesthesia. Br J Anaesth 2011; 106: 101- 11
26. Hans P, Verscheure S, Uutela K, Hans G, Bonhomme V: Effect of a fluid challenge on the Surgical Pleth Index during stable propofol-remifentanil anaesthesia. Acta Anaesthesiol Scand 2012; 56: 787-96
27. Bergmann I, Göhner A, Crozier TA, Hesjedal B, Wiese CH, Popov AF, Bauer M, Hinz JM: Surgical pleth index-guided remifentanil administration reduces remifentanil and propofol consumption and shortens recovery times in outpatient anaesthesia. Br J Anaesth 2013; 110: 622-8
28. Constant I, Nghe MC, Boudet L, Berniere J, Schrayer S, Seeman R, Murat I: Reflex pupillary dilatation in response to skin incision and alfentanil in children anaesthetized with sevoflurane: a more sensitive measure to noxious stimulation than the commonly used variables. Br J Anaesth 2006; 96: 614-9
29. Isnardon S, Vinclair M, Genty C, Hebrard A, Albaladejo P, Payen JF: Pupillometry to detect pain response during general anaesthesia following unilateral popliteal sciatic nerve block: a prospective, observational study. Eur J Anaesthesiol 2013; 30: 429-34
30. Larson MD, Behrends M: Portable infrared pupillometry: a review. Anesth Analg 2015; 120: 1242-53
31. Constant I, Sabourdin N: Monitoring depth of anesthesia: from consciousness to nociception. A window on subcortical brain activity. Paediatr Anaesth 2015; 25: 73-82
32. Luginbühl M, Bieniok C, Leibundgut D, Wymann R, Gentilini A, Schnider TW: Closed-loop control of mean arterial blood pressure during surgery with alfentanil: clinical evaluation of a novel model-based predictive controller. Anesthesiology 2006; 105: 462-70
33. Liu N, Chazot T, Hamada S, Landais A, Boichut N, Dussaussoy C, Trillat B, Beydon L, Samain E, Sessler DI, Fischler M: Closed-loop coadministration of propofol and remifentanil guided by bispectral index: a randomized multicenter study. Anesth Analg 2011; 112: 546-57
34. Orliaguet GA, Benabbes Lambert F, Chazot T, Glasman P, Fischler M, Liu N: Feasibility of closed-loop titration of propofol and remifentanil guided by the bispectral monitor in pediatric and adolescent patients: a prospective randomized study. Anesthesiology 2015; 122: 759-67
35. Le Guen M, Liu N, Tounou F, Augé M, Tuil O, Chazot T, Dardelle D, Laloë PA, Bonnet F, Sessler DI, Fischler M: Dexmedetomidine reduces propofol and remifentanil requirements during bispectral index-guided closed-loop anesthesia: a double-blind, placebo- controlled trial. Anesth Analg 2014; 118: 946-55
36. Liu N, Lory C, Assenzo V, Cocard V, Chazot T, Le Guen M, Sessler DI, Journois D, Fischler M: Feasibility of closed-loop co-administration of propofol and remifentanil guided by the bispectral index in obese patients: a prospective cohort comparison. Br J Anaesth 2015; 114: 605-14
37. Pasin L, Nardelli P, Pintaudi M, Greco M, Zambon M, Cabrini L, Zangrillo A: Closed- Loop Delivery Systems Versus Manually Controlled Administration of Total IV Anesthesia: A Meta-Analysis of Randomized Clinical Trials. . Anesth Analg 2016
38. Brogi E, Cyr S, Kazan R, Giunta F, Hemmerling TM: Clinical Performance and Safety of Closed-Loop Systems: A Systematic Review and Meta-Analysis of Randomized Controlled Trials. Anesth Analg 201
