# Supplementary material for: Clinical efficacy and safety of automatic remifentanil administration based on Analgesia Nociception Index monitoring during burn surgery under propofol anesthesia: A randomized controlled clinical trial
Source: PLoS One. 2025 May 5;20(5):e0322384. doi: 10.1371/journal.pone.0322384 (PMC12052174; doi:10.1371/journal.pone.0322384)
Supplement: Attachment — Submitted filename: RESPONSE TO REVIEWERS(3).docx [file pone.0322384.s011.docx]

Dear reviewers,

We thank you for your help in improving the quality of our manuscript. You’ll find our detailed answers below, with the corresponding changes in the manuscript. Modified parts of the manuscript have been highlighted for transparency.

In view of your questions, two additional bibliographic references have been added.

Sincerely

**Reviewer #1**: Thank you for reporting your findings.

Abstract: in the results, what quantity is the total decision for the estimates? State it is 95% confidence intervals or interquartile range the first time it is shown in the abstract if this is the case.

Thank you for pointing this lack of precision. Results are expressed in median[1^st^ ; 3^rd^ quartile). This has been notified in the abstract, at the end of the method. “A p value < 0.05 was considered statistically significant. Data are presented as median [1^st^ to 3^rd^ quartile].”

**Reviewer #2**:

Dear author(s)

First I congratulate you for your successful, well designed randomized controlled study entitled ‘Clinical efficacy and safety of automatic remifentanil administration based on Analgesia Nociception Index monitoring during general anesthesia: A randomized controlled clinical trial’.

We thank you for your kind assessment of our work.

However I have some comments.

1) The title accurately reflects the focus of the study, describing the intervention (automatic remifentanil administration), the context (based on the Analgesia Nociception Index), and the study design (a randomized controlled clinical trial) but could include the patient population or surgical context (e.g., burn surgery) to further enhance specificity.

Thank you for pointing the lack of precision. The title has been changed for: “Clinical efficacy and safety of automatic remifentanil administration based on Analgesia Nociception Index monitoring during burn surgery under propofol anesthesia: A randomized controlled clinical trial”

2) The abstract highlights the study's main findings, including reduced remifentanil dosage and maintained safety. It effectively communicates the significance of findings, especially the applicability of ANI monitoring. But you can provide additional context on the potential broader applications of the technology to strengthen the abstract's impact.

Thank you. The conclusion has been amended as you suggested: “Automatic remifentanil administration demonstrated good clinical performances during propofol anesthesia for burn surgery. It is likely that these results can be extrapolated to any surgical setting under general anesthesia, but this needs to be tested with further randomized clinical trials.

3) The introduction outlines the need for improved nociception monitoring and provides background on ANI. It justifies the study by referencing the limitations of current methods and describes the novel ANI-REMI-LOOP system. I think, this section is well-written, with clear context on the problem and study rationale.The hypothesis and objectives are well-stated, aligning with the study design. You can add a brief mention of how this research contributes to existing literature or fills identified gaps.

Thank you. The introduction has been enriched: “Several authors have described automatic opioid delivery during general anesthesia, but the lack of a clear physiological nociceptive signal has led to indirect assessments of nociception, often based on pharmacodynamic interaction models, hemodynamic reactions, or electroencephalographic signals related to cortical arousal [19–21]. In most studies, automated delivery of propofol and remifentanil was shown to be effective and safe, even if the delivery control strategies were rather simplistic, based on proportional, integral, derivative (PID) controllers, or controlling two pharmacological compounds (typically hypnotics and opioids) with a single input variable derived from a simplified EEG signal (e.g. BiSpectral® index) which is neither sensitive nor specific of nociception. In order to improve the performance of remifentanil automatic administration, we have recently designed an expert system for the automatic administration of remifentanil during general anesthesia, whose decision rules for modifying remifentanil flow rate are based on two objectives: i) maintaining stable hemodynamics by avoiding hypertension or tachycardia related to nociception as well as avoiding hypotension and bradycardia and ii) maintaining a relative predominance of paraS activity as assessed by ANI by dynamically increasing or decreasing the remifentanil infusion rate [22, 23].”

4) The objectives are stated indirectly within the introduction, focusing on assessing the efficacy and safety of ANI-based remifentanil administration. The study’s objectives are clear but could be outlined in a dedicated section for ease of reference. Please include a standalone section for explicitly stated objectives to improve structure and accessibility.

Thank you. A standalone section at the end of the introduction section has been added: “The objectives of the study were to evaluate the potential benefit of automatic remifentanil administration on remifentanil or propofol consumption, hemodynamic stability, ANI stability and postoperative pain. “

5) The methodology is robust, describing randomization, ethical considerations, intervention specifics, and outcome measures. Statistical analyses are detailed and appropriate for the study design. However, you have to provide additional details on how external validity was ensured, particularly for generalizing findings to other surgical types.

Thank you. Additional details have been provided at the beginning of the Material & methods chapter with three methodological references conducted with three different nociception monitors. “Several randomized clinical trials have been carried out in various surgical settings to evaluate the benefit of guiding the administration of remifentanil with nociception/antinociception balance monitors [16-18]. The designs and objectives of these studies are relatively standardized, we therefore designed the methodology of our clinical trial on the existing literature.”

6) The results demonstrate a significant reduction in remifentanil use and improved hemodynamic stability in the automatic group, without compromising safety or postoperative outcomes. Data is presented clearly with adequate use of tables and figures. In this section also you can emphasize clinical relevance of findings to make the benefits more relatable for practitioners.

Thank you. The clinical relevance of the results has been further underlined. “The median total remifentanil amount was significantly lower in the expert-system automatic group at 0.125 µg.kg-1.min-1 vs. 0.152 µg.kg-1.min-1 in the standard practice group, with a large effect size (standardized difference = –0.88), corresponding to 17.8% dose reduction (Table 2). The significant reduction in opioid delivery most probably resulted from the specific algorithm fine-tuning remifentanil delivery to the dynamic changes of ANI. Furthermore, the automatic group exhibited significantly reduced time spent with hemodynamic reactivity while other secondary outcomes showed no significant differences between groups (Table 3).”

7) The discussion contextualizes the findings within existing literature, emphasizing the benefits and potential limitations of ANI-based automatic remifentanil administration.The discussion of ANI’s potential in broader surgical settings is particularly strong. But please elaborate on alternative explanations for findings and explore how automation might reduce anesthetist workload.

Thank you. The discussion section has been enriched as follow: “Our findings can probably be explained by the capability of the electronic controller to dynamically adapt ("fine tune") opioid delivery to ANI monitoring. The mere number of remi flow rate changes during surgery in the automatic group compared with the number of target changes in the standard practice group gives an idea of the superiority of automation over manual target setting (57 [24–100] in the expert-system automatic group vs 7 [4.5–14]). The superiority of automation over manual administration has been extensively discussed by Coekelenbergh et al. The authors describe automatic and closed-loop devices for the various sensors routinely used during surgical procedures under anesthesia provided by hypnotic drugs, myorelaxants, opioids as well as fluids and vasopressors. The authors stress that automation is safe, reduces the workload of simple repetitive tasks while enhancing compliance with protocols and help improve care [32].”

8) The conclusions summarize the study's key findings, highlighting safety, efficacy, and potential for broader application. Please reiterate the significance of findings for clinical practice and patient outcomes.

Thank you. The conclusion has been changed accordingly: “The clinical efficacy and safety of an expert system based on ANI monitoring (MDoloris technology) for the automatic administration of remifentanil during propofol anesthesia has been demonstrated in a monocentric RCT, leading to significantly reduced remifentanil administration and less hemodynamic events. Pain and analgesic requirements in PACU at 2-h follow-up were not different between the groups. Automatic delivery of an optimal dose of remifentanil at precisely the right time using the ANI-REMI-LOOP device could allow each nociceptive stimulus to be controlled with a minimum dose of opioid while reducing the incidence and duration of hemodynamic events.”

9) For Table 1, please consider reformatting the table to separate demographic and clinical characteristics from procedural data for better readability. Also you can add a column for p-values where applicable, comparing the control and automatic groups for baseline variables.

Thank you. Table 1 has been edited accordingly, clearly separating “demographic data”, “clinical data”, “procedural data” and “Analgesic long-term medications secondary to burns”

Regarding the suggestion to provide p-value for between group differences in baseline characteristics, we respectfully disagree in light of the CONSORT recommendation: baseline characteristics should not be compared between groups in randomized controlled trial (RCT) but interpreted regarding the descriptive analysis “Item 15 from CONSORT : Such hypothesis testing is superfluous and can mislead investigators and their readers. Rather, comparisons at baseline should be based on consideration of the prognostic strength of the variables measured and the size of any chance imbalances that have occurred”. As commented by Altman DG, performing a significance test to compare baseline variables in RCT is to assess the probability of something having occurred by chance when we know that it did occur by chance (Altman DG. Comparability of randomized groups. The Statisticien (1985), pp 125-136).

10) For Table 2, please add a column explicitly showing the percentage reduction in remifentanil usage between groups for easier clinical interpretation and include a note clarifying whether the ITT and PP results differ due to excluded patients (e.g., patients with major protocol deviations).

Thank you. An additional column has been added, showing that the percentage reduction is the same following ITT and PP results. It is our belief that excluded patients did not impact the presented results.

11) For Table 3, draw attention to the most clinically significant differences (e.g., time spent in hemodynamic reactivity) using bold font or separate headings. A brief note summarizing the main findings of this table could help contextualize the results. For example, “The automatic group exhibited significantly reduced time in hemodynamic reactivity, but other outcomes showed no significant differences.”

Thank you. Two additional lines have been added to Table 3, indicating the number of hypotensive and bradycardic events. The following text has been added for clarity: “Twenty-one “Hemodynamic reactivity, hypotension, or bradycardia” events occurred in 12 cases (46.2%) in the expert-system automatic group and 27 “Hemodynamic reactivity, hypotension, or bradycardia” events occurred in 8 (30.8%) cases in the standard practice group (p = 0.25). The median duration for one event was 2 [2-3] min for the expert-system automatic group and 2 [1-6] min for the standard practice group (p=0.038), and their cumulative duration was 2 [2-3] versus 10.5 [3.75-25] min respectively (p=0.005). Two hypotension and no bradycardic events were observed in the expert-system automatic group whereas 4 hypotension and 3 bradycardic events occurred in the standard practice group (Table 3).”

12) Please emphasize in the manuscript text the potential clinical benefits of trends toward lower pain scores in the automatic group, even if not statistically significant. Discuss whether the pain score differences at later time points (e.g., H0 + 1h, H0 + 1h 45min) are likely clinically meaningful. Also you can include a brief note on how adverse events were assessed and whether the single case of nausea in the automatic group may be attributable to the intervention.

Thank you. The following sentences have been added in the discussion section: “Patients presented with similar pain levels after surgery, and analgesic requirements were also similar in both groups. The trend toward lower VAS from H0+45min in the automatic group was not statistically significant, and may have resulted from a lack of statistical power, as it was only a secondary endpoint. Side effects such as nausea and vomiting were infrequent in both groups."

**Reviewer #3**: The authors present the results of a randomized study conducted in patients scheduled for excision of burns and skin grafts. The aim of the study was to assess the safety and the efficacy of an innovative closed-loop system to automatically deliver remifentanil based on the continuous monitoring of hemodynamic parameters and of the ANI. The study is very original, and the results correspond to a proof-of-concept of the innovation tested. The manuscript is well written and easy to read. Figures and tables are of high quality and adequate. Nonetheless, the manuscript raised the following concern.

MAJOR CONCERNS

1. Methods: page 6: the closed-loop system defines as the 1st priority to protect the patient’s safety. The priority cannot be bypassed. That is a good thing for the patients, but it raises several concerns. This priority appears to be very close to the “old” management of anesthesia that was based on the fluctuations of hemodynamic parameters to adapt the infusion rate of anesthetic drugs. Considering that point, what is the added value of using ANI to monitor intraoperative analgesia?

Thank you for raising this question. The safety focus has led us to prioritize hemodynamics (priority 1) over antinociception (priority 2). As presented in the result section, hemodynamic impairment was infrequent in both groups, and represented a small proportion of anesthesia duration when present, so that regulation of remifentanil’s flow was primarily driven by ANI measures (priority 2 rules). We have added two short explanations in the general description of the algorithm: “This rule has been implemented in order to limit the risk of severe hypotension (SBP<70 mmHg): it only allows limiting the infusion flow in case of SBP decrease.” and “Priority 2 rules: dynamic adaptation of remifentanil flowrate. Several studies have demonstrated that variations in ANI enable to anticipate hemodynamic reactivity in relation with the NAN balance. We therefore implemented ANI based decision rules in order to anticipate and possibly avoid hemodynamic reactivity by increasing the infusion flow in order to maintain ANIm slightly over ANImin. Other rules also aimed at decreasing the infusion flow in order to maintain ANI bellow ANImax. .”

2. Eligible patients were burned patients. How many of these patients were treated with beta-blockers to reduce the hypermetabolic response? Could the treatment with beta-blockers impact the measurement of the ANI?

Thank you for raising this point. Our unit mainly uses beta-blockers in patients with Burn Surface Area over 10-15%. No patient included in the presented RCT benefited from beta-blocker treatment for this purpose.

3. Page 7: exclusion criteria: how did you define “sustained tachycardia” and “impaired blood pressure”?

Thank you. We have added the definition of "Sustained tachycardia and impaired blood pressure" in the anesthesia protocol section : " Baseline HR>120 bpm, baseline SBP<75 mmHg or baseline SBP>160 mmHg led to exclusion. During anesthesia, sustained tachycardia (HR > 120 bpm), impaired blood pressure (SBP<75 mmHg) or prolonged hypertension (SBP>160 mmHg) despite adequate appliance of the anesthetic protocol led to exclusion from the per-protocol analysis.

4. Methods: page 8-9: the protocol of induction of anesthesia differs between the 2 groups. Could the authors explain how they proceed to define this protocol for induction and comment on the potential impact of this difference in the management of patients on the results of the study? In particular, the remifentanil dose for induction appears to be higher in the manual group.

Thank you for raising this point. Our unit is well accustomed with ANI monitoring and has been using ANI for remifentanil Ce manual guidance for more than 10 years. We merely described the standard practice of our unit for the "standard practice group" of the presented manuscript. When designing the algorithm for expert-rules automatic remifentanil administration, the point of using either Minto pK/pD model for flow rate changes (which would lead to an indirect relation between the actual flow rate and ANI monitoring) or using directly the administered flow rate as expressed in µg.kg-1.min-1 was solved mostly in regard of the expected extrapolability to any kind of surgical and anesthesia practices. Our experience with ANI monitoring on the other hand gave us confidence that NAN balance variations were indeed similar in this population as with any surgical patient population.

We have tried to convey these elements in the discussion section of the presented manuscript : " Second, our unit always proceed during induction of anesthesia as we described in the method section for the standard practice group: remi Ce target set at 6 ng.ml until orotracheal intubation, then lowered to 3 and adapted to maintain the ANIm in the [50-70] target. As the amount of administered remifentanil would depend on the duration of the induction procedure, in particular the ease of tracheal intubation, the resulting amount of remifentanil administered during the induction phase of anesthesia could not precisely be anticipated. On the other hand, the "automatic group" needed an easily implemented remifentanil induction protocol, for which we had experience from the early phase of remifentanil clinical availability : a 1 µg.kg-1 bolus was standard procedure before TCI was available. Similarly, 0.1 µg.kg-1.min-1 infusion dose after induction was considered enough for situations when nociception would be low, typically before the start of any surgical procedure. Considering the whole duration of anesthesia, around 90 min, it was our belief that the difference in method would not bias the results. Extrapolation of the "algorithm for automation" was also of concern: using simple calculations and dosage requirements based on the patient’s ideal body weight enables other teams in various surgical settings and various patient populations to reproduce our method. "

5. Methods: one of the main concerns is the protocol of anesthesia in the manual group. How did the authors proceed to define this protocol of management? What was the experience of the investigators in this anesthetic protocol (learning curve)? The sample size is quite small with 25 patients in each group, and it could have been difficult to adequately apply a new anesthetic protocol in patients included in the study. Did the authors measure the good applicability of the anesthetic protocol and the rate of deviance to the anesthetic protocol? Please comment that points and their potential impact on the results of the study.

Thank you. As expressed in our answer to question 4, our unit is well accustomed to the "standard practice" procedure.

6. Results: page 12: what is the clinical relevance of the intergroup difference observed on the intraoperative remifentanil dose? In particular, the results suggest that it had no impact on early postoperative analgesia. Is the conclusion of the study: “no matter with how intraoperative analgesia is managed, the main concern is hemodynamics”?

Thank you. As answered before (reviewer 2, Q6), a column has been added to table 2, and the following text has been added in the result section : “The median total remifentanil amount was significantly lower in the expert-system automatic group at 0.125 µg.kg-1.min-1 vs. 0.152 µg.kg-1.min-1 in the standard practice group, with a large effect size (standardized difference = –0.88), corresponding to 17.8% dose reduction (Table 2). The significant reduction in opioid delivery most probably resulted from the specific algorithm fine-tuning remifentanil delivery to the dynamic changes of ANI”

+++ajouter les quelques phrases expliquant qu(on a juste une tendance sur la douleur post-op, possiblement du à un manqué d’effectif.

7. Results: what is the potential impact on prognosis to decrease the cumulative time of hemodynamic reactivity, hypotension or bradycardia? The results of the outcome are presented as percentage of time spent, but it could be informative to have these results as absolute time spent with hemodynamic reactivity, hypotension or bradycardia in min, and the duration of the longer episode of hemodynamic reactivity, hypotension or bradycardia in min in each group. This supplemental data would help to assess the clinical relevance of this result.

Thank you. This additional information have added in Table 3, as well as the following sentence in the result section : " Furthermore, the automatic group exhibited significantly reduced time spent with hemodynamic reactivity while other secondary outcomes showed no significant differences between groups (Table 3).”

and

Twenty-one “Hemodynamic reactivity, hypotension, or bradycardia” events occurred in 12 cases (46.2%) in the expert-system automatic group and 27 “Hemodynamic reactivity, hypotension, or bradycardia” events occurred in 8 (30.8%) cases in the standard practice group (*p* = 0.25). The median duration for one event was 1 [1-2] min for the expert-system automatic group and 2 [1-6] min for the standard practice group (p=0.038), and their cumulative duration was 2 [2-3] versus 10.5 [3.75-25] min respectively (p=0.005). Two hypotension and no bradycardic events were observed in the expert-system automatic group whereas 4 hypotension and 3 bradycardic events occurred in the standard practice group (Table 3).

8. Figure 3: the difference observed on hemodynamics seems to be mainly related to bradycardia. Is this correct? If yes, one explanation could be that investigators in the manual group could have been more concern by hypotension than tachycardia, especially when the decrease in heart rate did not reach critical thresholds. Please comment that point.

Thank you for pointing this out. Figure 3 presents HR and SBP in both groups, as median, first and third quartiles. The overlap between the two groups is clear, but readers may follow the median values at each Measuring Point and wrongly infer a difference between groups, which was not statistically nor clinically present. Table 3 presents the various hemodynamic "alterations" among the two groups, with additional details.

Moreover, as presented in Table 3, bradycardia only occurs in 3/26 patients in the standard practice group and in 0/26 patients in the automatic group.

9. In the automatic group, the investigator was assisted by an engineer to manage intraoperative administration of remifentanil. Did the authors think that the presence of an additional member of the anesthesia team (the engineer) in the operating room could have impacted the quality of intraoperative administration of remifentanil? Please comment.

Thank you. As the tested device was experimental (hence the clinical trial), the presence of the engineer was for security only : he assessed continuously whether the automatic device was working properly (which it did), and would have alerted the anesthetist in charge in case of any electronical (sensors and connections), mechanical (electrical syringe) or software (bug, critical or not) impairment. We have added the following sentence in the Method section " An investigator-engineer (J.D.J.) was present only to guarantee the patients’ safety in the event of a malfunction of the ANI-REMI-LOOP software. The engineer did not act on the management of anesthesia nor influence the decisions of the anesthetists (M.J. or M.Hu.). "

10. Table 3: please reformulate the 2nd line: “time spent relative to intervention duration”. What “time spent” refers to?

Thank you for point this out. We have renamed this line: "duration relative to intervention duration (%)"
